# Ribonuclease MCPiP1 contributes to the loss of micro-RNA-200 family members in pancreatic cancer cells

**DOI:** 10.18632/oncotarget.26310

**Published:** 2018-11-13

**Authors:** Françoise Boudouresque, Carole Siret, Aurélie Dobric, Françoise Silvy, Philippe Soubeyran, Juan Iovanna, Dominique Lombardo, Yolande Berthois

**Affiliations:** ^1^ Aix-Marseille Univ, INSERM UMR 911, CRO2, Marseille, France; ^2^ Aix-Marseille Univ, CNRS, INSERM, CIML Marseille, France; ^3^ Present address: Aix-Marseille University, INSERM, CNRS, Institut Paoli-Calmettes, CRCM, Pancreatic Cancer Team, Marseille, France

**Keywords:** pancreatic cancer, micro-RNA-200, MCPiP1, dicer1

## Abstract

The microRNA-200 (miR-200) family is frequently down-regulated in tumors, including pancreatic adenocarcinomas (PDACs). In this study we have examined the mechanisms involved in the loss of miR-200s in tumoral pancreatic cells. Whereas miR-200 gene promoters appear methylated in mature miR-200 deficient cell lines, miR-200 precursors are detected in nuclear but not cytoplasmic compartment of these cells, indicating that promoter hypermethylation is not sufficient to explain the deficit of mature miR-200s. The ribonuclease Monocyte Chemotactic Protein-induced Protein-1 (MCPiP1) may counteract Dicer1 in miRNA maturation process. MCPiP1/Dicer1 mRNA and protein ratios appear higher in miR-200 deficient compared to miR-200 proficient cells, suggesting that MCPiP1 may compete with Dicer1 in mature miR-200 deficient cells. Inhibition of MCPiP1 allows the detection of miR-200 precursors in cytoplasm of miR-200 deficient cells, confirming its involvement in the loss of miR-200s. Also, reversion of MCPiP1/Dicer1 ratio by over-expression of Dicer1 in miR-200 deficient cells leads to the recovery of mature miR-200s. Finally, whereas human malignant pancreatic tissues (PDACs) express lower miR-200 levels than non malignant tissues (non-MPDs), MCPiP1/Dicer1 ratio appears higher in PDACs, when compared to non-MPDs, supporting the hypothesis that MCPiP1/Dicer1 ratio is determinant in regulating miR-200 maturation process in a subset of tumoral pancreatic cells.

## INTRODUCTION

Ductal adenocarcinoma of the pancreas (PDAC) is the fourth leading cause of cancer death and has the poorest survival rate for any solid cancer [1]. Complete surgical resection represents the only curative treatment option but late initial diagnosis due to disease unspecific symptoms prohibits resection. Moreover, current therapy resistance and the early metastatic spread of pancreatic cancers account for therapy inefficacy, and high mortality-to-incidence ratio emphasizes the need for novel targets in therapy and diagnosis [2].

MicroRNAs (miRNAs) are short non-coding RNAs that play an important role in development, normal physiology, and diseases. MiRNAs are transcribed as long primary transcripts, pri-miRNAs, by RNA polymerase II. The pri-miRNAs are then processed in the nucleus by the microprocessor complex constituted of the rinonuclease Drosha [3], its regulatory subunit DiGeorge Critical Region 8 (DGCR8) [4, 5] and various cofactors [6–8]. Microprocessor cleaves pri-miRNAs to generate 70–100 nucleotide long pre-mature miRNAs (pre-miRNAs) [4]. Pre-miRNAs are then exported to the cell cytoplasm by Exportin 5 (XPO_5_) along with the cofactor RanGTP [9, 10] where they are further cleaved by a complex composed of the ribonuclease III enzyme Dicer and the double-stranded RNA binding protein TRBP2, generating short miRNA duplexes [11, 12]. After degradation of one strand, the mature single strand miRNAs are loaded onto Argonaute proteins within the RNA-induced silencing complex (RISC) [13] and guided to complementary sequences in the 3′UTR of target mRNAs to repress their translation.

In cancer in particular, miRNAs play essential functions in regulating tumor progression, metastasis and response to radio- and chemo-therapy [14]. Although some miRNAs have been shown to be upregulated and to play oncogenic roles, most are repressed in cancers relative to normal tissue counterparts [15]. Among the many miRNAs reported to be repressed in cancer cells, the miR-200 family is thought to play special role in human health and disease. The miR-200 family can be divided into two clusters: the miR-200b,a,429 cluster located on chromosome 1p36 and containing miR-200b, miR-200a, and miR-429. The second cluster contains miR-200c and miR-141 and is located on chromosome 12p13 [16]. The miR-200 family was first described as major regulator of the epithelial-mesenchymal transition (EMT) process. Thus, the miR-200 family members directly target a number of genes involved in various aspects of EMT [17, 18], in particular the cadherin-1/E-cadherin transcriptional repressors ZEB1 and ZEB2. This leads to the restoration of an epithelial phenotype, characterized by an increase in cadherin-1/E-cadherin expression, and decreased cell migration and invasion [19]. Consistent with their anti-oncogenic function, the expression of the miR-200 family members was reported to be impaired in a variety of human tumors [20–22]. Moreover, their expression level was shown to be correlated with tumor clinical outcome in breast, colorectal, ovarian and prostate cancers, and proposed as a prognostic marker for cancer patients with bladder and gastric cancers [23–27]. In PDACs, alterations in the expression of miR-200s have also been reported and are thought to contribute to tumor progression, invasion and response to therapy [28]. Thus, the tumor suppressive function of miR-200 family is supported by a number of clinical studies that reported under-expression of different miR-200 members in PDACs [29, 30]. Also, miR-200s were shown to function by regulating EMT in pancreatic tumoral cells. Thus, over-expression of miR-200s had an inhibitory effect on human pancreatic cancer stem cells by deregulating EMT-related genes *in vitro* and *in vivo* [31, 32]*.* These events were associated with decreased colony formation, invasion, chemoresistance and xenograft growth in *nude* mice [32]. Furthermore, low level of miR-200s was correlated with low survival rate for PDAC patients [29, 30]. Importantly, miR-200 family is also thought to play an essential role in drug-resistance of pancreatic cancer cells. Thus, Li *et al.* [33] showed that the expression of miR-200 family was significantly down-regulated in gemcitabine (GEM)-resistant cells and re-expression of miR-200 family resulted in increased cell response to GEM. Moreover, miR-200 expression in primary tumor xenografts of patient-derived pancreatic cancers carrying wild type epidermal growth factor receptor was correlated with response to erlotinib *in vivo* [34].

The expression of miR-200s may be repressed through different mechanisms. Like protein-coding genes, numerous miRNA genes in human cancers are located in chromosomal regions that frequently exhibit amplification, deletion or translocation. Thus, down-regulation of miR-200b,a,429 gene in human hepatocellular carcinomas has been shown to be attributable, at least in part, to genome deletion [35]. Changes in miR-200 expression level can also occur through both transcriptional and post-transcriptional mechanisms. In particular, whereas the miR-200 family is known to exert tumor suppressor activity by silencing ZEB1 and ZEB2, ZEB1 has been reported to down-regulate miR-200 expression in the context of a mutual repression loop, in breast, colon and pancreas cancers [36, 37]. Moreover, in KRAS-driven cancer including PDACs, miR-200 expression was suppressed by KRAS activation [38]. This suppression, mediated by ZEB1, promoted cell survival and EMT in pancreatic cancer cell lines. Also, mucin1 (MUC1), a transmembrane glycoprotein overexpressed and associated to a bad prognostic in PDACs, was shown to be involved in miR-200 repression through its interaction with ZEB1 [39]. Transcriptional silencing of miRNA has also been linked to epigenetic regulation such as methylation or histone modifications of miRNA genes. The regulatory regions of both miR-200 clusters contain CpG-rich sequences and several studies have shown that silencing of miR-200 genes in a large variety of cancers, such as colon, breast, lung and pancreas cancers, is concomitant with hypermethylation of the CpG islands [40, 41]. More recently, the focal adhesion protein Kindlin 2 was found to form a complex with DNA (cytosine-5-)-methyltransferase 3 alpha (DNMT3A) in breast cancer cells to induce CpG island hypermethylation of the miR-200 promoter, leading to the decreased expression of the miR-200 family members [42]. In addition to DNA methylation, histone modification has also been described to impact the expression of the miR-200 family. Thus, Lim *et al.* (2013) found that in immortalized human mammary epithelial cells, the miR-200b,a,429 cluster was silenced primarily through polycomb group-mediated histone modifications, whereas the miR-200c,141 cluster was repressed via DNA methylation [40]. At last, reduced levels of mature miRNAs may also result of defects in their biogenesis pathway. In particular, impairment in the nuclear export of pre-mature miRNA forms has been reported in a number of human primary tumors. Thus, numerous miRNA precursors, including miR-200 precursor forms, were found to be retained in the nucleus of cancer cells in pancreas and liver tumors [43], and the presence of XPO_5_ inactivating mutations in a subset of human tumors was shown to be involved in trapping of miRNAs precursors in the nucleus [44]. Additionally, lower Dicer1 expression level found in a variety of human tumors [45, 46] is though to be responsible of the loss or decreased level of a number of miRNAs. Their biogenesis also depends on complex post-transcriptional processing controlled by a number of binding proteins such as Lin-28, heterogeneous nuclear ribonucleoprotein A1 (hnRNPA1) and KH-type splicing regulatory protein (KHSRP) [47]. Furthermore, changes in the level of several miRNAs has also been shown to depend on the activity of the ribonuclease MCPiP1 that functions as a direct antagonist to Dicer1, leading to cleavage and degradation of pre-miRNAs in cytoplasm compartment [48].

Despite a number of data highlighting the important roles of miR-200 family in cell growth, metastasis, EMT, and drug resistance in pancreatic cancer cells, the molecular mechanisms responsible of the down-regulation of miR-200 family members in these cells have been poorly examined and remain obscure. In this context, clarifying the mechanisms that lead to the loss of miR-200s in PDACs appears essential. In this study we demonstrate that in addition of gene promoter methylation, the level of miR-200 family members in a subset of tumoral pancreatic cells is impacted by the protein ratio MCPiP1/Dicer1.

## RESULTS

### The expression of miR-200 family members in tumoral pancreatic cell lines correlates with GEM responsiveness

Altered expression of miR-200 family members has been described in PDACs. In this context, the expression of miR-200 family members (mature forms) was measured in pancreatic cell lines by RT-qPCR. The expression levels reported in Table 1 indicate that all miR-200 family members were significantly expressed in BxPC3, Capan-2, Soj-6 and HPDE cell lines. Conversely, miR-200 expression levels were considerably lower in Mia-Paca2 and PANC1 cells. In the following of this manuscript, BxPC3 and Soj-6 cell lines will be referenced to as miR-200 proficient cells, and Mia-Paca2 and PANC1 cells as miR-200 deficient cells.

**Table 1:** Expression level of miR-200 family members in tumoral pancreatic cell lines

Cell lines	miR-200a/snU6 ×100	miR-200b/snU6 ×100	miR-429/snU6 ×100	miR-200c/snU6 ×100	miR-141/snU6 ×100
BxPC3	115.0 ± 24.5	41.7 ± 12.6	10.6 ± 1.67	121 ± 14.8	15.2 ± 2.6
Soj-6	43.9 ± 2.75	14.7 ± 0.34	10.27 ± 0.09	63.3 ± 8.33	28.17 ± 4.57
HPDE	20.1 ± 1.9	7.95 ± 1.1	0.79 ± 0.06	76.5 ± 19	4.09 ± 0.89
Capan-2	31.2 ±3.6	11.7 ± 3.3	1.22 ± 0.03	43.8 ± 9.9	4.21 ± 0.45
Mia-Paca2	0.036 ± 0.026	0.014 ± 0.0029	0.05 ± 0.004	0.076 ± 0.01	0.012 ± 0.0009
PANC1	0.058 ± 0.051	0.028 ± 0.013	0.012 ± 0.0014	0.052 ± 0.016	0.012 ± 0.0014

The expression of miR-200s was determined by RT-qPCR as indicated in Materials and Method section and normalized with snU6 gene expression. Data are the mean ± SD of 3 separate experiments.

In order to verify whether the presence of miR-200s may be correlated to GEM responsiveness, the antitumoral effect of GEM was evaluated *in vitro*, on the tumoral pancreatic cell lines. As shown in Table 2, variable cell growth inhibitory effects of GEM were observed, depending on the tumoral cell lines examined. Whereas Mia-Paca2 and PANC1 cells were little affected by GEM (IC_50_ = 150 ± 12 nM and 200 ± 21 nM, respectively), BxPC3, Capan-2 and Soj-6 cell lines displayed higher sensitivity to GEM, with IC_50_ of 12 ± 4 nM, 46 ± 8 nM and 30 ± 8 nM, respectively. Similarly, GEM was shown to affect the proliferation of normal epithelial pancreatic cells HPDE with IC_50_ = 20 ± 4 nM. These data confirm that the growth inhibitory effect of GEM may be associated to miR-200 expression level in tumoral pancreatic cells.

**Table 2:** Growth response of tumoral pancreatic cell lines to gemcitabine (GEM)

Cell lines	GEM IC_50_, nM
Mia-Paca2	150 ± 12
PANC1	200 ± 21
BxPC3	30 ± 8
Soj-6	12 ± 4
HPDE	20 ± 4
Capan-2^*^	46 ± 8

Cells were treated for 5 days in the absence or in the presence of different doses of GEM. Cell proliferation was assessed by MTT metabolism then IC_50_ determined. Values are the mean ± SD of 2 separate experiments. ^*^, because Capan-2 cells display very low double time (94 hours), treatment with GEM was extended to 16 days.

### MiR-200 overexpression in tumoral pancreatic cell lines decreases EMT marker expression

**Figure 1:**
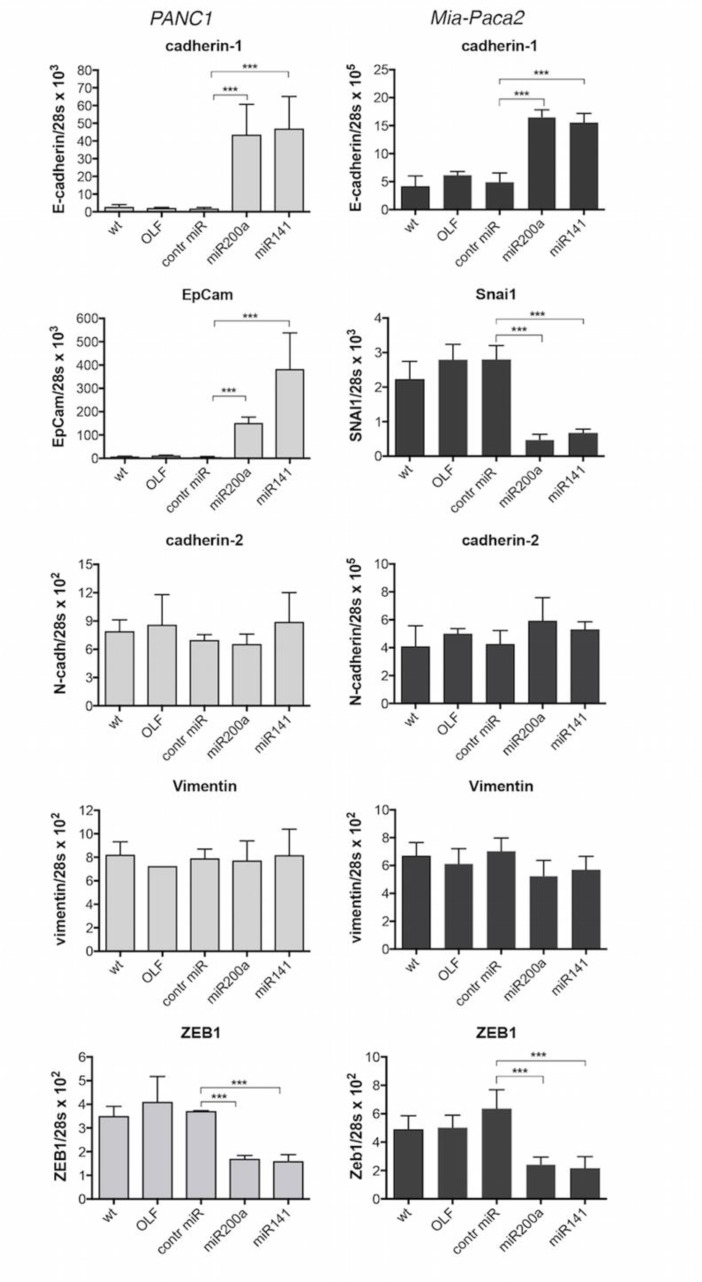
Effect of miR-200 over-expression on the expression of EMT and epithelial markers in PANC1 and Mia-Paca2 cells PANC1 (grey columns) and Mia-Paca2 (black columns) cells were transfected with synthetic miR-200a or miR-141 oligonucleotides in the presence of the transfection reactif oligofectamine (OLF). RNA was then extracted two days post-transfection and gene expression was measured by RT-qPCR. Control cells received oligofectamine in the absence (OLF) or in the presence of control miRNA oligonucleotide (control miR). Values are the mean ± SD of triplicates. Data are representative of 2 separate experiments. ^***^P < 0.001.

EMT is known to play an especially important role in the development and progression of PDACs. A number of reports have shown that the miR-200 family plays a crucial role in the control of EMT in cancer cells, through transcriptional repression of EMT-inducible genes. In order to verify the involvement of miR-200s in EMT regulation in tumoral pancreatic cells, the expression of a number of epithelial (cadherin-1/E-cadherin, EpCam) and mesenchymal (cadherin-2/N-cadherin, vimentin, ZEB1, Snai1) markers was measured following over-expression of miR-200s (Figure 1). Whereas cadherin-2/N-cadherin and vimentin expression was not modified, transfection of both Mia-Paca2 and PANC1 cells with miR-200a or miR-141 resulted in a moderate but significant decrease of ZEB1 expression level, when compared to control oligonucleotide-transfected cells. On the contrary, forced expression of miR-200s led to an important increase of cadherin-1/E-cadherin and EpCam expression level in PANC1 cells. Although lower than in PANC1 cells, an increased expression of cadherin-1/E-cadherin, associated with a diminution of Snai1 expression level, was observed in Mia-Paca2 cells transfected with miR-200 oligonucleotides.

### MiR-200 precursor forms are detectable in mature miR-200 deficient tumoral pancreatic cell lines

**Figure 2:**
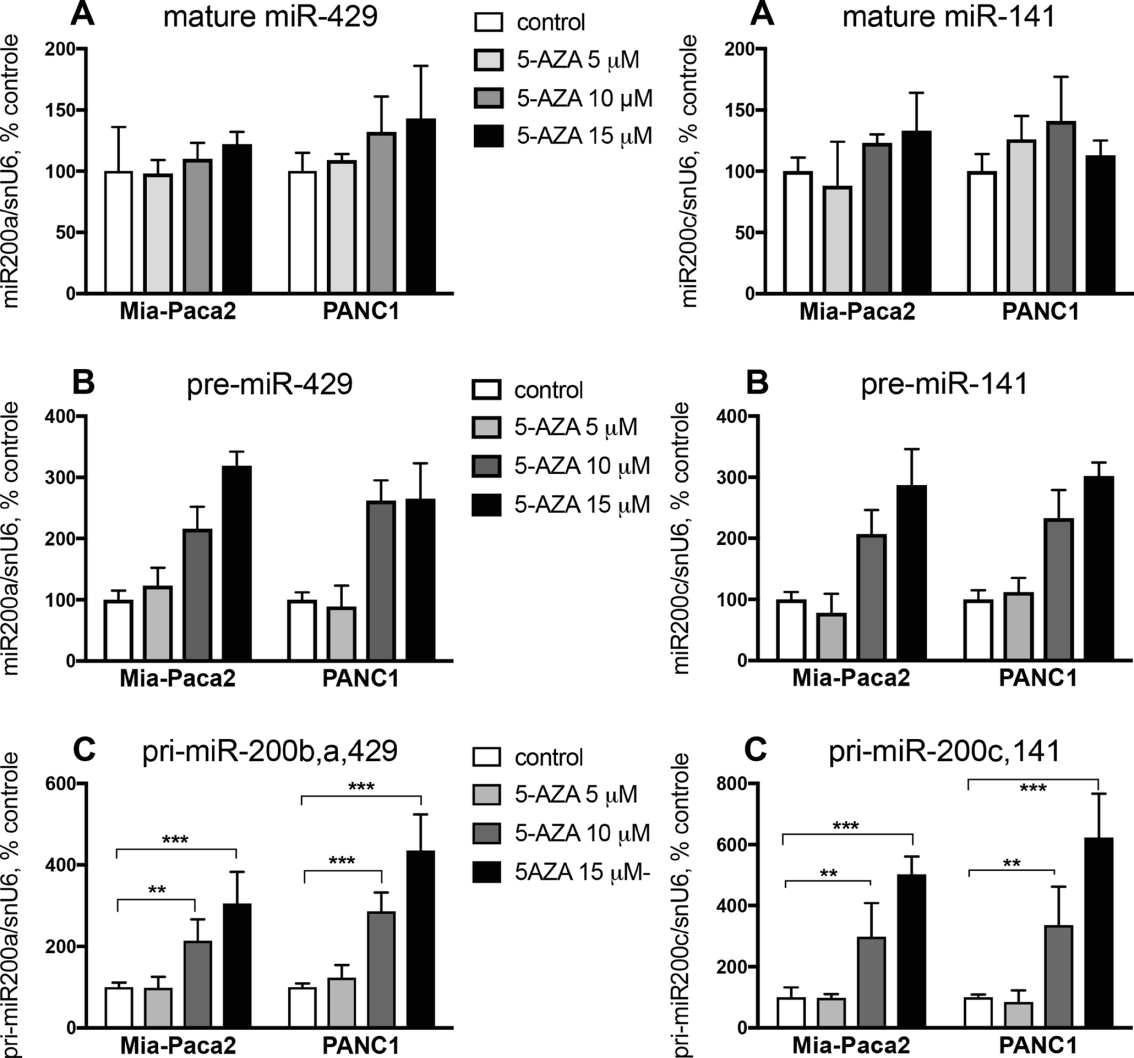
Effect of 5-AZA on miR-200 expression level in Mia-Paca2 and PANC1 cells Cells were treated for 2 days in the absence or in the presence of different concentrations of 5-AZA. At the end of the treatment, RNA was extracted and the expression of mature (miR-429, miR-141) (**A**), pre-mature (pre-miR-429, pre-miR-141) (**B**) and primary (pri-miR-200b,a,429, pri-miR-200c,141) (**C**) miR-200s was measured by RT-qPCR. Values are the mean ± SD of triplicates. Data are representative of 2 separate experiments. ^**^P < 0.01; ^***^P < 0.001.

Epigenetic modifications have been described to affect gene expression in cancer cells. In particular, the expression of a number of miRNA may be altered *via* methylation and/or deacetylation of their promoters. When methylation status of miR-200b,a,429 and miR-200c,141 gene promoters was examined, only methylated promoters were detectable in both Mia-Paca2 and PANC1 cell lines (Supplementary Figure 1A, 1B), suggesting that promoter methylation prevents miR-200 gene transcription and thus leads to the loss of mature miR-200s in these cells. Nevertheless, while unmethylated gene promoters were detected following treatment of Mia-Paca2 and PANC1 cells with the demethylant agent 5-Azacytidine (5-AZA) (Supplementary Figure 1C, 1D), measurement of miR-200 expression showed that 5-AZA failed to restore significant levels of mature miR-429 and miR-141 (Figure 2A). These data indicate that gene promoter methylation is not the only mechanism involved in miR-200 deficiency observed in these cells.

The presence of miR-200 precursors in Mia-Paca2 and PANC1 cells was then verified by RT-PCR. In order to differentiate between both primary and pre-mature precursors, low molecular weight (LW) RNAs were separated by electrophoresis of total RNA on acrylamide gel, then extracted (Supplementary Figure 2A, 2B). Unexpectedly, RT-PCR performed on LW fractions indicated the presence of pre-miR-141 in both miR-200 -proficient (BxPC3 and Soj-6) and -deficient (Mia-Paca2 and PANC1) cells. Also, primary miR-200c,141 (pri-miR-200c,141) could be detected in total but not in LW RNA preparations obtained from Mia-Paca2 and PANC1 cells (Supplementary Figure 2C, 2D). Furthermore, treatment of Mia-Paca2 and PANC1 cells with 5-AZA resulted in a significant and dose-dependent increase of the level of primary forms of miR-200s (pri-miR-200b,a,429 and pri-miR-200c,141) as evidenced by RT-PCR analysis (Supplementary Figure 2E). Likely, quantitative measurement of miR-200 expression showed that 5-AZA increased the expression of pre-miR-429 and pre-miR-141 to 300% of untreated control cells. Similar results were observed when measuring the expression of the primary miR-200 forms (pri-miR-200b,a,429 and pri-miR-200c,141) (Figure 2B, 2C). All together, these data indicate that i) promoter methylation impairs only partially miR-200 gene transcription in Mia-Paca2 and PANC1 cells, and ii) increasing miR-200 gene transcription and precursor processing with 5-AZA fails to restore significant levels of mature miR-200s, strongly suggesting that a default in maturation process occurs in these cells and contributes to the loss of mature miR-200s.

### MiR-200 pre-mature forms are present in nuclear but not cytoplasmic compartment of mature miR-200 deficient tumoral pancreatic cell lines

**Figure 3:**
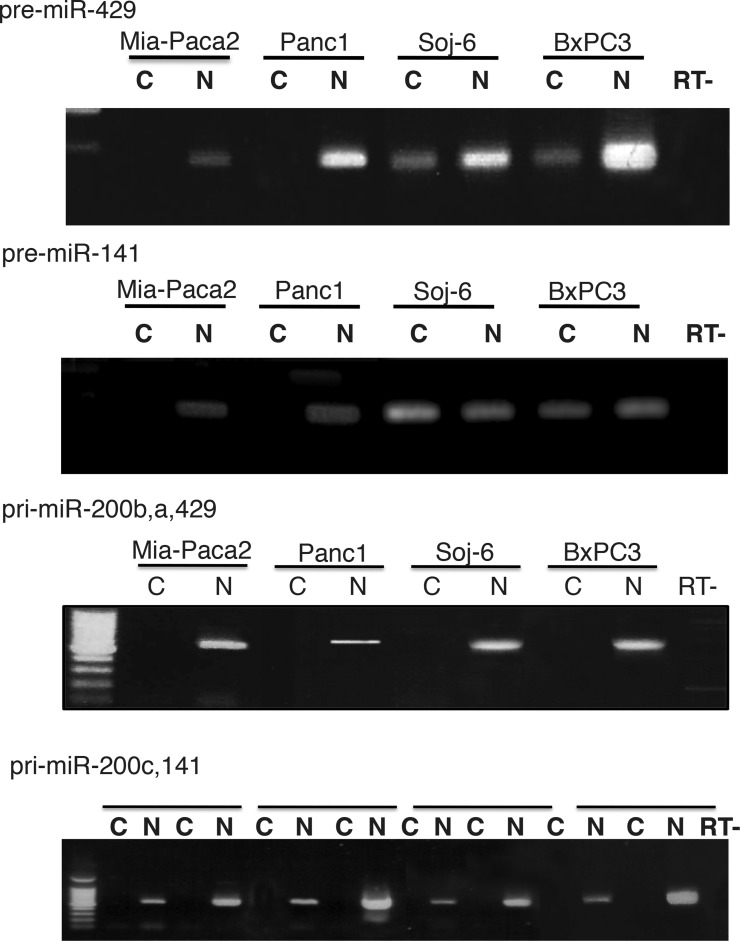
Subcellular location of miR-200 precursors in tumoral pancreatic cell lines Total RNA was extracted from nuclear (N) and cytoplamic (C) fractions prepared from tumoral pancreatic cells. Low molecular weight (LW) RNAs were obtained by separation of total RNA on 8% acrylamide gel. Primary miR-200s (pri-miR-200b,a,429 and pri-miR-200c,141) were amplified from total RNA. Pre-mature miR-200s (pre-miR-429 and pre-miR-141) were amplified from LW RNAs. Note that primers for primary forms do not amplify pre-mature miR-200s. Data are representative of 2 separate experiments. RT-, negative reverse transcription.

MiRNA primary forms produced in nuclei are cleaved to generate pre-mature miRNAs that are then exported in cytoplasm where they undergo maturation process. To compare subcellular location of miR-200 precursors in mature miR-200 deficient Mia-Paca2 and PANC1 cells *vs* miR-200 proficient BxPC3 and Soj-6 cells, the presence of primary and pre-mature miR-200s was examined by RT-PCR in nuclear and cytoplasmic cell compartments. Quality of cell fractions was verified by the presence of cytoplasmic 5S ribosomal RNA and small nuclear U6 (snU6) RNA (Supplementary Figure 3). Primary and pre-mature miR-200s were amplified from total and LW RNA fractions, respectively. As expected, miR-200b,a,429 and miR-200c,141 primary forms were exclusively detected in nuclear compartment of all the cell lines examined (Figure 3). Whereas pre-mature miR-429 and miR-141 were present in both nuclei and cytoplasm of BxPC3 and Soj-6 cell lines, pre-mature miR-200s were confined in nuclear compartment of Mia-Paca2 and PANC1 cells. Measurement of precursor expression level by quantitative RT-PCR confirmed the presence of both primary and pre-mature miR-200s forms in nuclear but not cytoplasmic compartments of Mia-Paca2 and PANC1 cells (Table 3).

**Table 3:** Expression level of miR-200 precursors in tumoral pancreatic cell lines

	miR-200s/snU6 × 10^2^							
	Mia-Paca2		PANC1		BxPC3		Soj-6	
	C	N	C	N	C	N	C	N
pri-miR-200b,a,429	ND	1.8 ± 0.60	ND	3.1 ± 0.71	ND	210 ± 57	ND	390 ± 49
pri-miR-200c,141	ND	2.5 ± 0.39	ND	3.3 ± 0.84	ND	410 ± 13	ND	660 ± 81
pre-miR-429	ND	0.15 ± 0.07	ND	0.28 ± 0.11	18 ± 6.3	41 ± 9.2	0.83 ± 0.22	64 ± 19
pre-miR-141	ND	0.33 ± 0.09	ND	0.32 ± 0.09	23 ± 3.8	27 ± 7.5	0.63 ± 0.24	110 ± 21

The expression of primary (pri-miR) and pre-mature (pre-miR) miR-200s was determined by RT-qPCR as described in Material and Methods section, and normalized with snU6 gene expression. Data are the mean ± SD of 2 separate experiments. C, cytoplasmic compartment; N, nuclear compartment; ND, no detectable.

### The loss of mature miR-200s in miR-200 deficient tumoral pancreatic cell lines is not due to impaired function of XPO_5_

**Figure 4:**
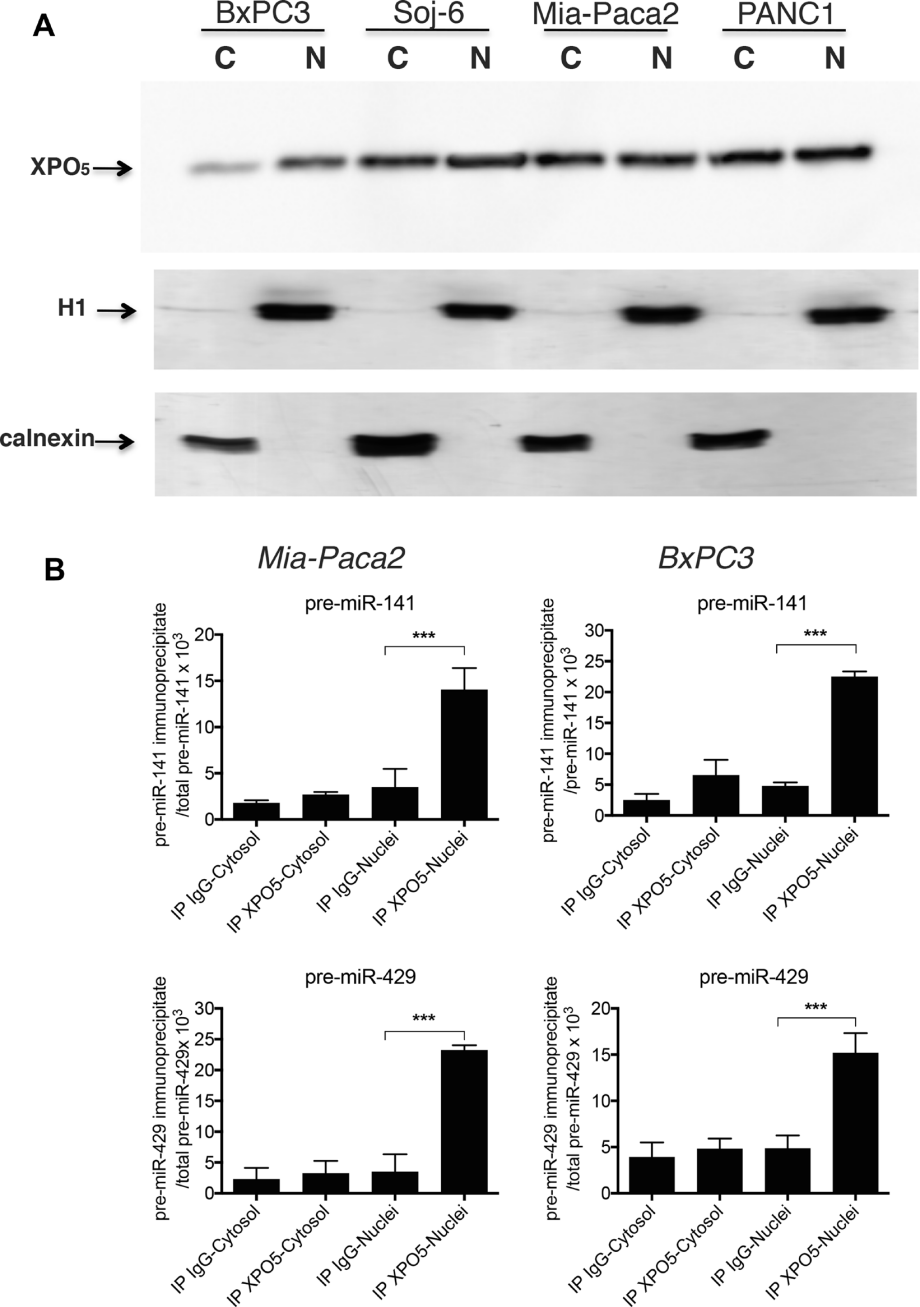
Co-immunoprecipitation of pre-mature miR-200s with XPO_5_ in tumoral pancreatic cell lines (**A**) nuclear (N) and cytoplasmic (C) fractions prepared from tumoral pancreatic cell lines were separated by electrophoresis on acrylamide gel and XPO_5_ detected by immunoblotting. Cytoplasmic and nuclear preparations were verified by immunoblotting of calnexin and histone H1, respectively. Data are representative of 2 separate experiments. (**B**) XPO_5_ was immunoprecipitated in cytoplasmic and nuclear fractions prepared from Mia-Paca2 and BxPC3 cells. Premature miRNAs that co-immunoprecipitate with XPO_5_ were extracted then measured by RT-qPCR. In parallel, total pre-mature miRNAs level present in cytoplasmic and nuclear fractions was determined prior to immunoprecipitation. As control, immunoprecipitation with irrelevant IgG was performed. Each value is the mean ± SD of triplicates. Data are representative of 2 separate experiments. Data are expressed as the ratio between immunoprecipitated and total pre-mature miRNA determined for each cell fraction. pre-miR-141, pre-mature miR141; pre-miR-429, pre-mature miR429.

The absence of pre-miR-200s into cytoplasm of Mia-Paca2 and PANC1 cells could be due to a default in molecular interaction between XPO_5_ and pre-miR-200s. Data reported in Figure 4A showed that similar levels of XPO_5_ were present in both nuclear and cytoplasmic compartments of all cell lines examined. XPO_5_-bound pre-miR-200s were then measured by RT-qPCR after immuno-precipitation of XPO_5_ in nuclear and cytoplasmic fractions of Mia-Paca2 and BxPC3 cells. As expected, pre-miR-429 and pre-miR-141 co-immunoprecipitated with XPO_5_ in nuclear fractions of BxPC3. Although the level of pre-miR-200s measured in Mia-Paca2 cell nuclei was significantly lower than in BxPC3 cells (cf Table 3), we were able to co-immunoprecipitate both pre-miR-141and pre-miR-429 with XPO_5_ in nuclear fraction of Mia-Paca2 cells (Figure 4B), confirming that XPO_5_ is able to bind pre-mature miR-200 forms in these cells.

**Figure 5:**
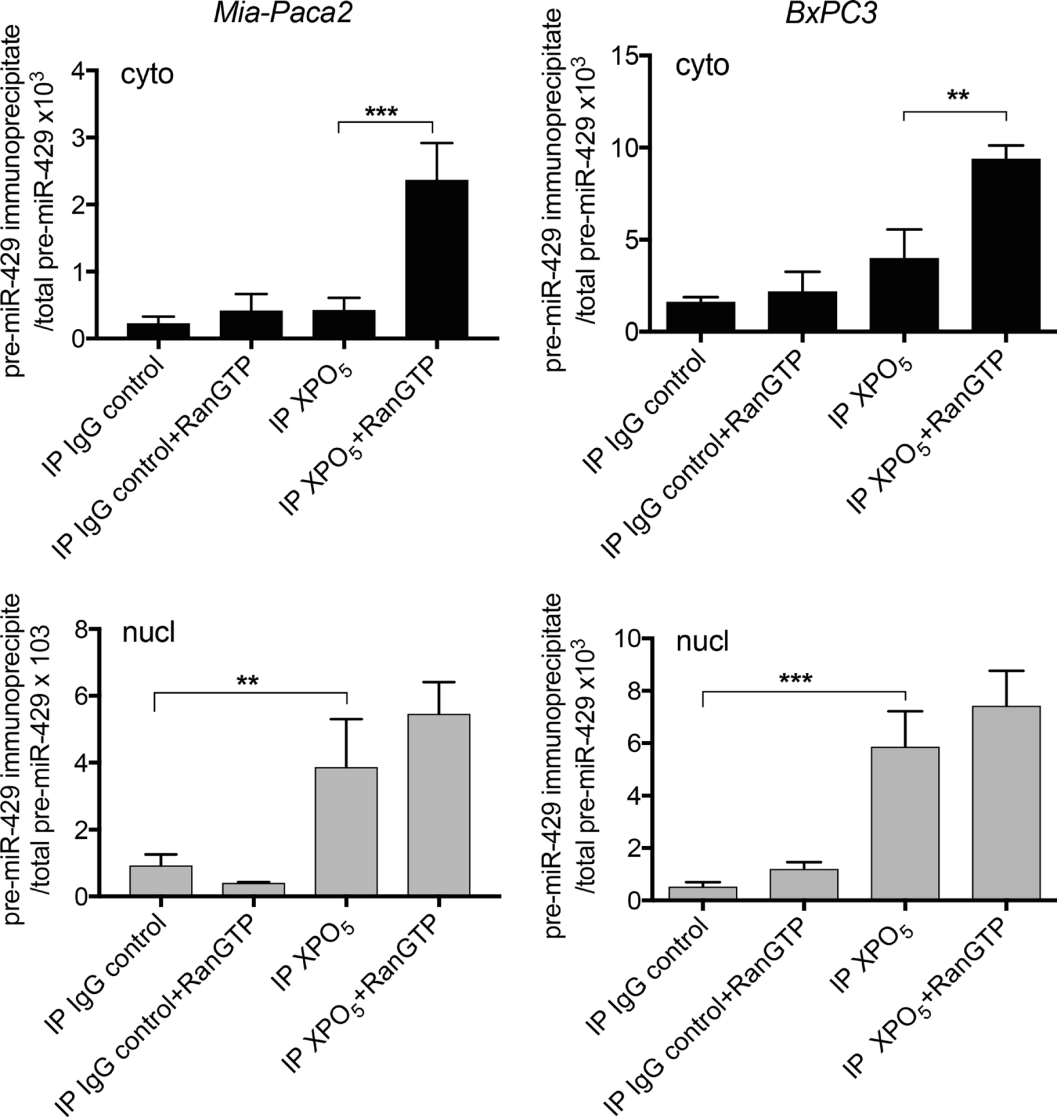
Effect of RanQ69L-GTP on the interaction between XPO_5_ and pre-miR-200s in tumoral pancreatic cell lines BxPC3 and Mia-Paca2 were pre-treated with 2 μM RanQ69L-GTP for 1 h at 37° C, before the preparation of cytoplasmic (cyto, black columns) and nuclear (nucl, grey columns) fractions. XPO_5_ was immunoprecipitated from each cell compartment and co-immunoprecipitated pre-mature miRNAs were extracted then measured by RT-qPCR. In parallel, total pre-mature miRNAs level was determined prior to immunoprecipitation in both cytoplasmic and nuclear fractions. As control, immunoprecipitation with irrelevant IgG was performed. Values are the mean ± SD of triplicates. Data are representative of 2 separate experiments and are expressed as the ratio between immunoprecipitated and total pre-mature miRNA determined for each cell fraction. pre-miR-429, pre-mature miR-429; RanGTP, RanQ69L-GTP. ^**^P < 0.01; ^***^P < 0.001.

Transport of XPO_5_/pre-miRNA complexes through nuclear pores requires its association with the cofactor Ran-GTP. Upon translocation into the cytoplasm, Ran-GTP hydolysis leads to the dissociation of the complex. To verify whether XPO_5_/pre-miR-200 complexes were able to move through nuclear membrane in Mia-Paca2 cells, specific interactions were increased and stabilized by treating cells with the non-hydrolysable RanQ69L-GTP. Co-immunoprecipitation of XPO_5_/pre-miR-429 was then performed in nuclear and cytoplasmic fractions. In the absence of RanQ69L-GTP, pre-miR-429 was co-immunoprecipitated with XPO_5_ in nuclear compartment of both BxPC3 and Mia-Paca2 cells (Figure 5). Conversely, pre-miR-429 could not be immunoprecipitated with XPO_5_ in cytoplasmic compartment of BxPC3, probably due to the rapid dissociation of the complex. Likely, pre-miR-429/XPO_5_ complexes were undetectable in Mia-Paca2 cytoplasmic extracts. However, in the presence of RanQ69L-GTP, significant levels of pre-miR-429 were co-immunoprecipitated with XPO_5_ in cytoplasmic compartment of Mia-Paca2 and BxPC3 cells, indicating that XPO_5_ is able to transport pre-miR-200s through nuclear membrane in both cell lines.

### MCPiP1/Dicer1 protein ratio impacts pre-miR-200 processing in mature miR-200 deficient tumoral pancreatic cell lines

**Figure 6:**
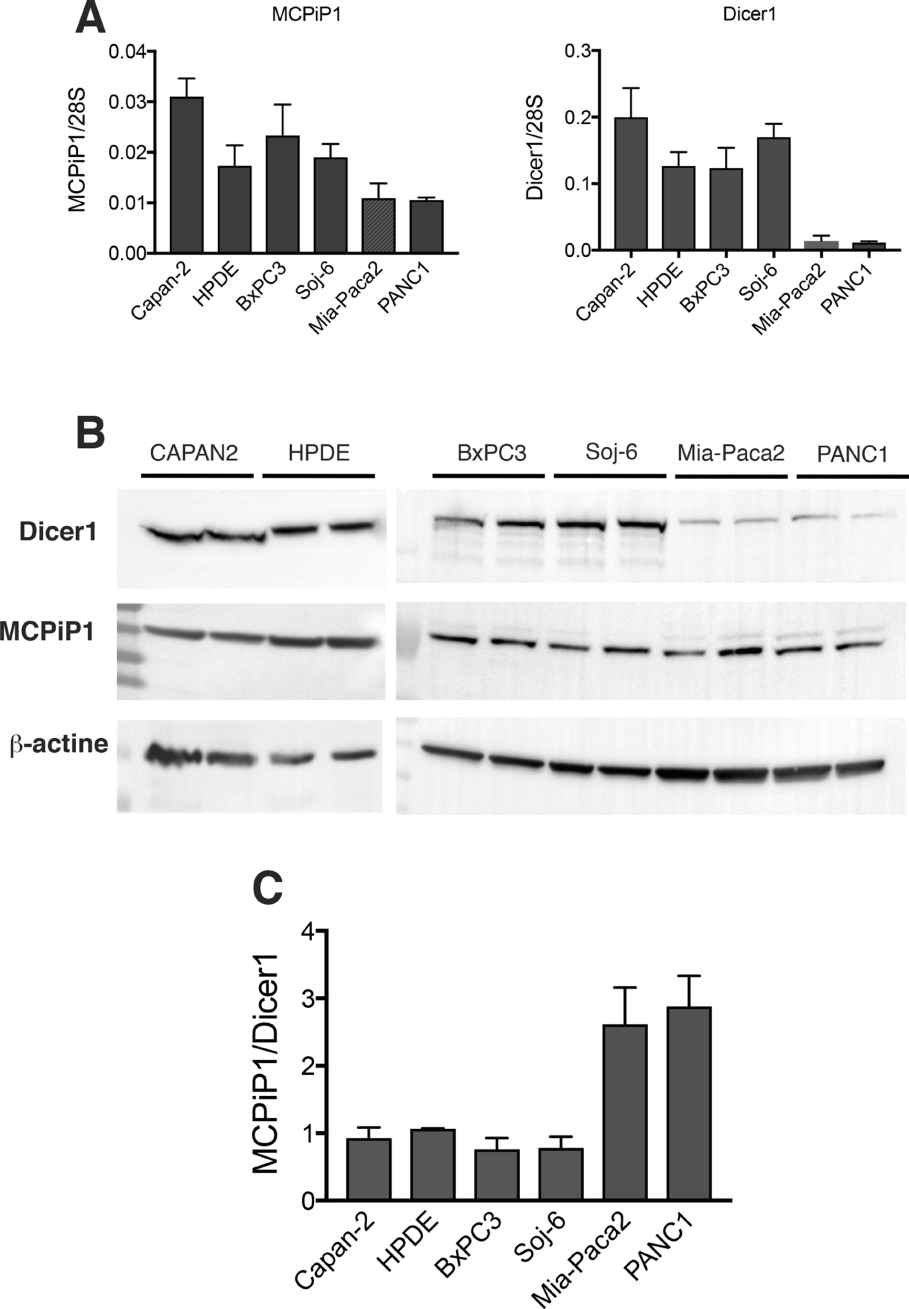
Expression of Dicer1 and MCPiP1 in tumoral pancreatic cell lines (**A**) MCPiP1 and Dicer1 gene expression was determined by RT-qPCR on RNA extracted from tumoral pancreatic cells. Data were normalized with the expression of the housekeeping gene 28S ribosomic RNA. (**B**) Dicer1 and MCPiP1 protein level was analysed by immunoblotting on cell lysates prepared from tumoral pancreatic cells. The integrated optical density of each band was quantified using ImageJ software. Values obtained for each sample were normalized for the corresponding β-actin protein level. (**C**) protein expression ratio between MCPiP1 and Dicer1 determined from immnublottings for all the cell lines examined.

It has been shown that multiple steps of miRNA biogenesis are regulated by several RNA binding factors. MCPiP1 has been described to be involved in miRNA degradation by competing with Dicer1 for pre-miRNA binding. The potential role of MCPiP1 in the loss of mature miR-200s in Mia-Paca2 and PANC1 cells was then evaluated. RT-qPCR measurements indicate that Mia-Paca2 and PANC1 cells expressed very low Dicer1 level when compared to miR-200 proficient cells (Capan-2, HPDE, BxPC3 and Soj-6 cell lines) (Figure 6A). Immunoblot analysis indicated the presence of one major isoform at 218 kDa in all cell lines examined. In agreement with the RT-qPCR data, Dicer1 protein expression level appeared considerably reduced in both Mia-Paca2 and PANC1 cell lines (Figure 6B). Whereas MCPiP1 expression measured by RT-qPCR was found to be slightly lower in Mia-Paca2 and PANC1 cells, MCPiP1 protein level was similar in all cell lines analyzed. However, the relative MCPiP1/Dicer1 protein ratio determined for each cell line, was much more elevated in Mia-Paca2 and PANC1 cells than in Capan-2, HPDE, BxPC3 and Soj-6 cells (Figure 6C), strongly suggesting that in miR-200 deficient cells, MCPiP1 could compete efficiently with Dicer1 for pre-miR-200 binding.

**Figure 7:**
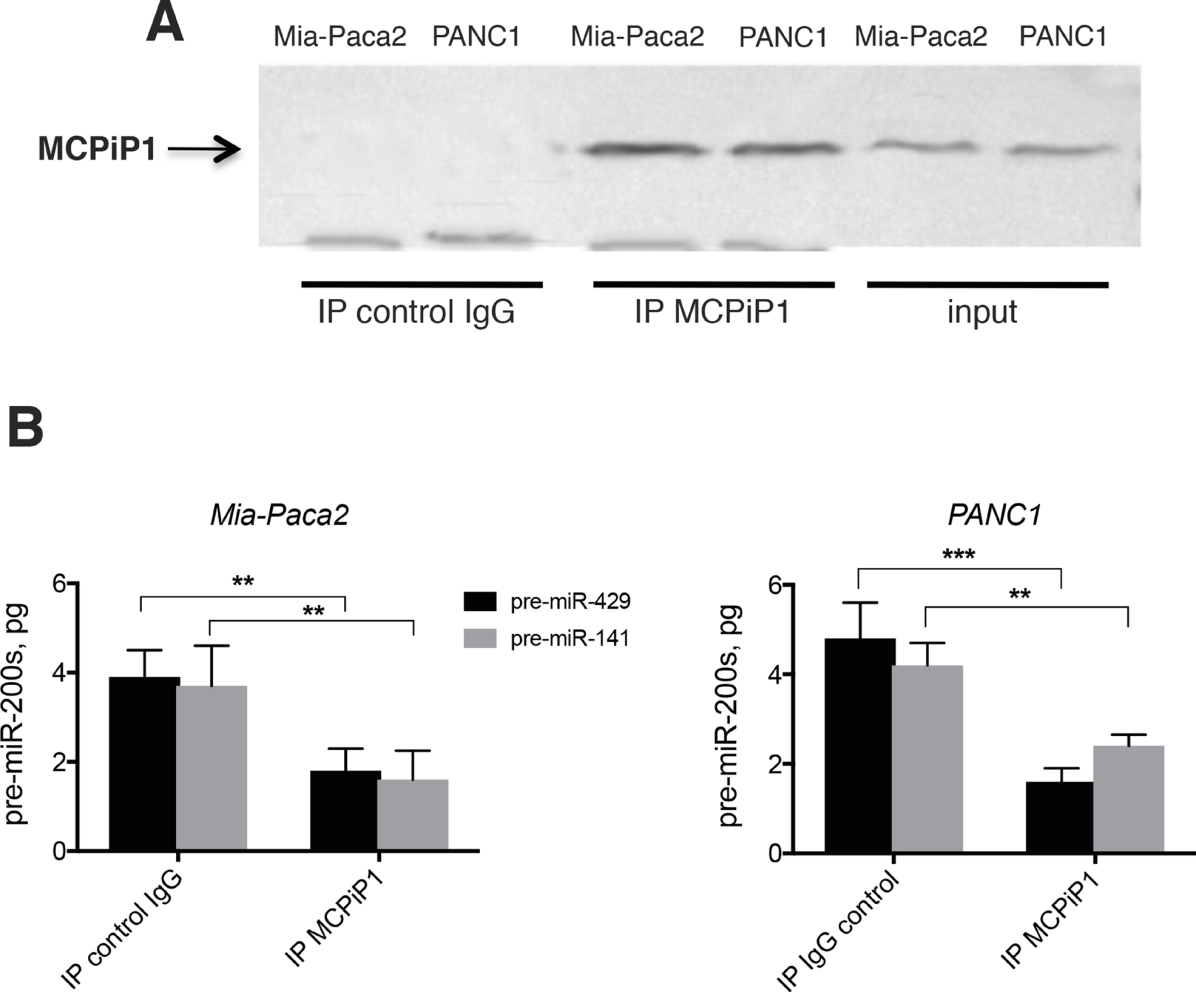
Effect of immunoprecipited MCPiP1 on the stability of pre-mature miR-200s (**A**) MCPiP1 was immunoprecipited in lysates prepared from Mia-Paca2 and PANC1 cells. (**B**) Immunoprecipitated MCPiP1 was incubated in the presence of 10 pg of synthetic pre-miR-200 oligonucleotides for 30 min at 30° C. Pre-miR-200s were then extracted and quantified by RT-qPCR. Pre-miR-429, pre-mature miR-429; pre-miR-141, pre-mature miR-141. Data are representative of 2 separate experiments. ^**^P < 0.01; ^***^P < 0.001.

In order to verify whether MCPiP1 might be involved in the loss of miR-200 in Mia-Paca2 and PANC1 cells, potential association between MCPiP1 and pre-miR-200 was first examined. For this purpose, pre-miR-200s were extracted and measured by RT-qPCR following MCPiP1 immunoprecipitation. As expected, pre-miR-200s could not be detected when MCPiP1 was immunoprecipitated from Mia-Paca2 or PANC1 cell lysates (not shown). This could be due, either to the sequestration of pre-miR-200s in nuclei, or to their rapid degradation by MCPiP1 in cytoplasmic compartment. However, when MCPiP1, immunoprecipitated from Mia-Paca2 or PANC1 cell lysates, was incubated in the presence of synthetic pre-mi-429 or pre-miR-141 oligonucleotides a diminution of detectable pre-miR-200s was observed (Figure 7), demonstrating that MCPiP1 issued from miR-200 deficient cells is able to degrade pre-mature miR-200 forms.

To confirm the involvement of MCPiP1 in the down-regulation of mature miR-200s, MCPiP1 was first under-expressed in Mia-Paca2 cells by transit transfection with MCPiP1 siRNA. Immunoblot analysis confirmed that MCPiP1 protein expression level was reduced in MCPiP1 siRNA transfected cells, compared to cells transfected with control scramble siRNA. Measurement of miR-200s by RT-qPCR shown that the inhibition of MCPiP1 expression led to a moderate but significant augmentation of the level of mature miR-200s in Mia-Paca2 and PANC1 cells (Supplementary Figure 4), demonstrating the involvement of MCPiP1 in the loss of miR-200s in tumoral pancreatic cells. Whether MCPiP1/Dicer1 ratio is determinant for miR-200 maturation, increasing Dicer1 protein level in Mia-Paca2 cells should lead to an augmentation of mature miR-200 level. Dicer1 was then over-expressed in Mia-Paca2 cells. Data show that Mia-Paca2 cells transfected with Dicer1 expression vector produced significant higher Dicer1 protein level, as compared to their counterpart cells transfected with the empty vector. Moreover, when compared to wild-type or vector transfected cells, a reversion of MCPiP1/Dicer1 ratio in favor of Dicer1 was observed in Dicer1 over-expressing Mia-Paca2 cells (Supplementary Figure 5).

**Figure 8:**
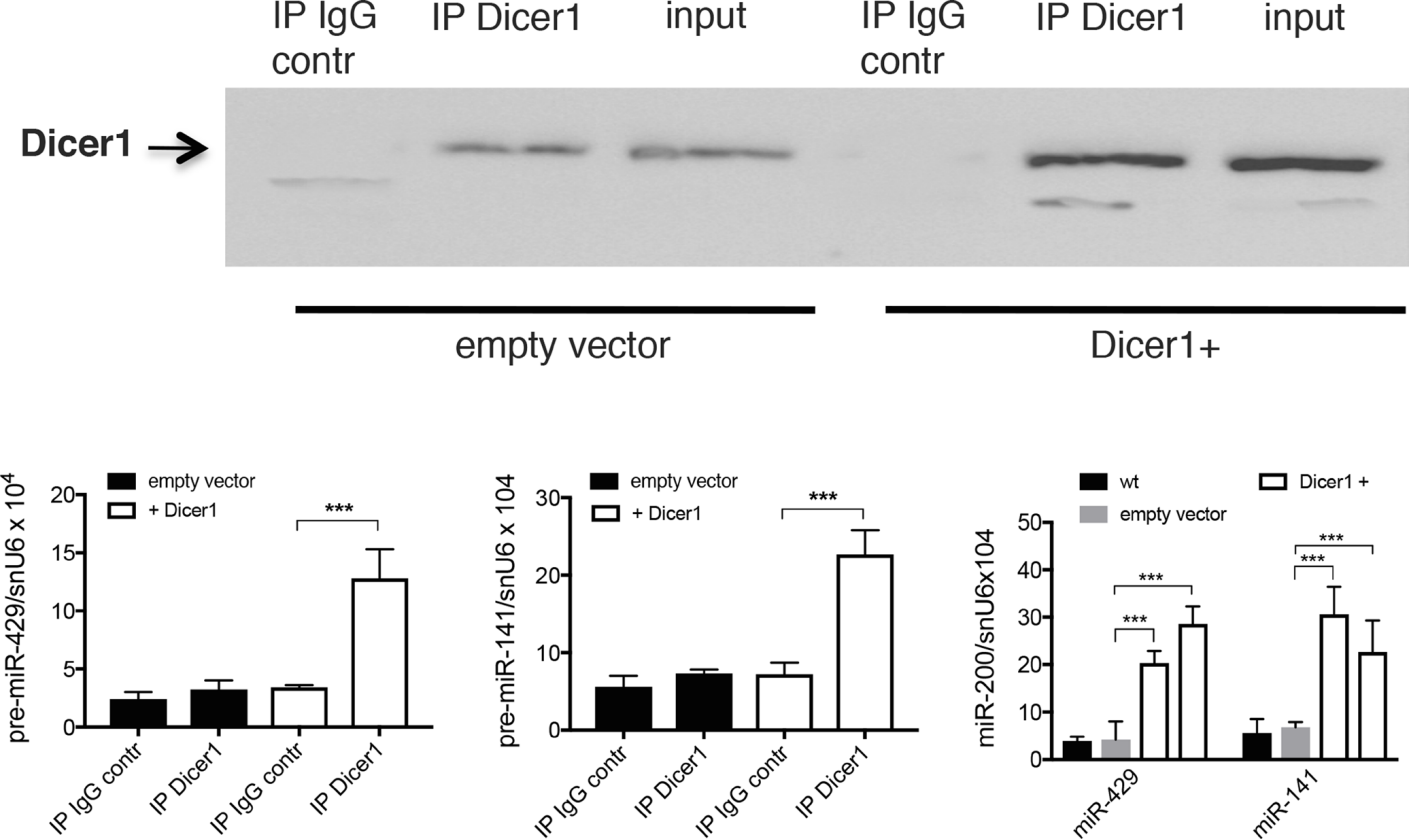
Effect of Dicer1 overexpression on the expression level of pre-miR-200s in Mia-Paca2 cells (**A**), Dicer1 was immunoprecipited from Dicer1 under-expressing (empty vector) and over-expressing (Dicer1+) MiaPaca2 cells. Irrelevant IgG was used as control (IgG contr). Co-immunoprecipited pre-miR-429 (**B**) and pre-miR-141 (**C**) were extracted and measured by RT-qPCR. (**D**), expression level of mature miR-429 and miR-141 in Mia-Paca2 cells under- and over-expressing Dicer1, measured by RT-qPCR. wt, wild type cells; Dicer1^+^, Dicer1 over-expressing cells. Data are representative of 2 separate experiments. ^***^P < 0.001.

To assess the effect of Dicer1 over-expression on pre-miR-200s level, co-immunoprecipitation was performed in Dicer1 over-expressing Mia-Paca2 cells. Whereas we were unable to detect significant amount of pre-miR-200s when Dicer1 was immunoprecipitated from control cells, data reported in Figure 8 show that both pre-miR-429 and pre-miR-141 co-immunoprecipitated with Dicer1 in Dicer1 over-expressing cells. Furthermore, over-expression of Dicer1 was associated with a significant increase of mature miR-200 expression. Thus, miR-429 and miR-141 levels were 5–6 folds more important in Dicer1 over-expressing Mia-Paca2 cells than in wild type or empty vector-transfected cells.

### High MCPiP1/Dicer1 expression ratio is associated to low miR-200 expression level in malignant pancreatic tissue

**Figure 9:**
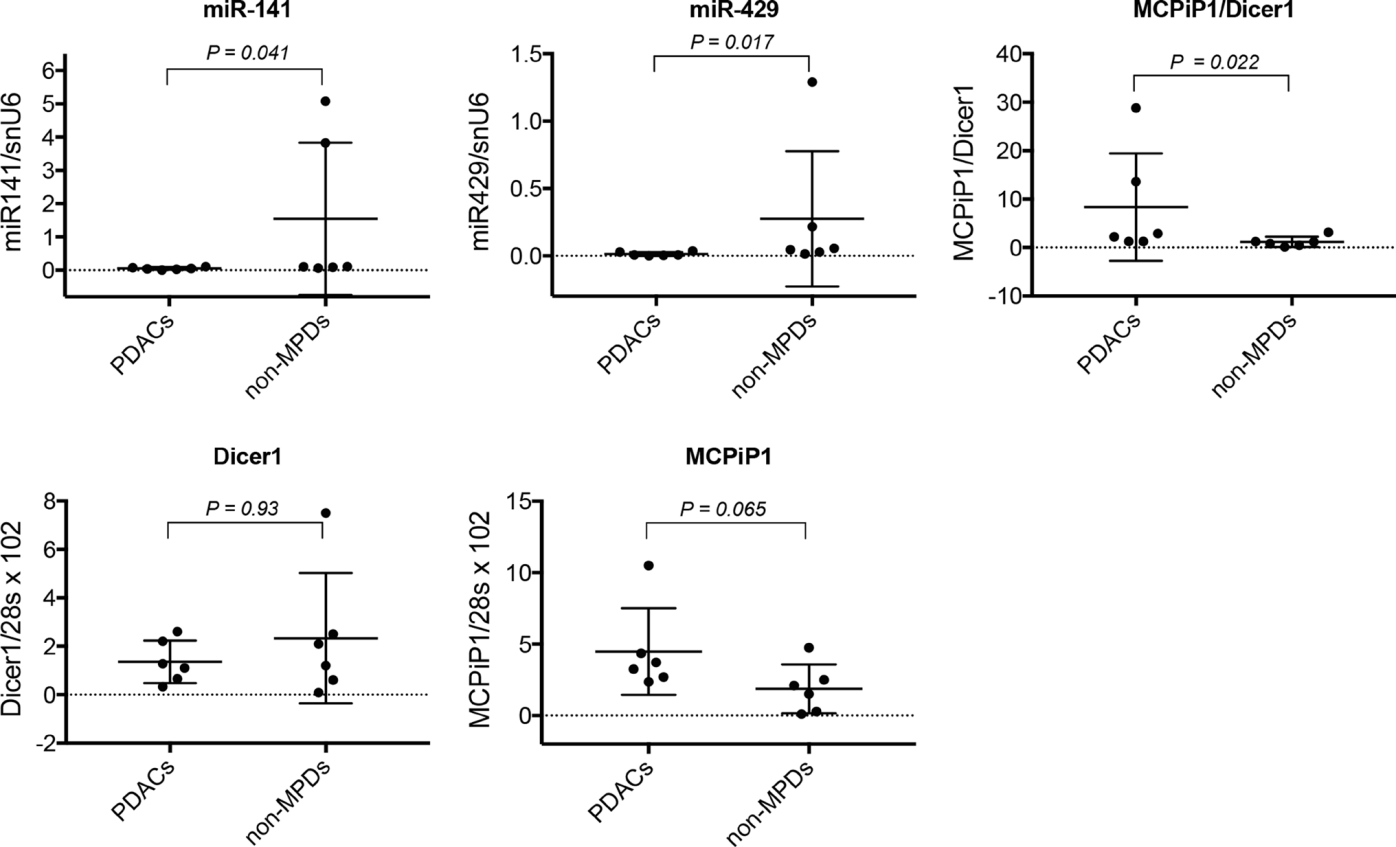
Analysis of miR-200 expression and MCPiP1/Dicer1 expression ratio in human pancreatic diseases The expression of MCPiP1, Dicer1, miR-141 and miR-429 was measured by RT-qPCR in malignant (PDACs, *n* = 6) and non-malignant (non-MPDs, *n* = 6) pancreatic diseases. MiR-141 and miR-429 expression was normalized with snU6 housekeeping gene expression. MCPiP1 and Dicer1 expression levels were normalized with the expression of 28s, then MCPiP1/Dicer1 ratio determined for each sample. For each group, median values and 5% and 95% confidence intervals for MCPiP1, Dicer1, MCPiP1/Dicer1 ratio, miR-141 and miR-429 are reported.

Since miR-200 expression level appeared to be influenced by MCPiP1/Dicer1 ratio in a subset of tumoral pancreatic cell lines, the potential association between miR-200 level and MCPiP1/Dicer1 ratio was examined in both malignant (PDACs) and non-malignant (non-MPDs) pancreatic tissue (Table 4). Whereas MCPiP1 and Dicer1 expression levels were found to be not significantly different between both sample groups, data reported in Figure 9 show that the ratio between MCPiP1 and Dicer1 expression was significantly higher in PDACs, compared to non-MPDs group (*P* = 0.022). Conversely, both miR-141 and miR-429 were shown to be less expressed in PDACs than in non-MPDs (*P* = 0.041 and *P* = 0.017, respectively), supporting the hypothesis that miR-200 expression in PDACs might be dependent, at least in part, on the relative expression of MCPiP1 and Dicer1.

**Table 4:** Clinical data for pancreatic tissues analyzed

Patients	Age^1^	Gender^2^	TNM^3^		
			T	N	M
PDAC 1	73	F	3	3	0
PDAC 2	48	F	0	0	0
PDAC 3	68	F	0	0	0
PDAC 4	69	M	9	9	1
PDAC 5	73	F	2	0	0
PDAC 6	80	M	3	1	0
CCP 1	74	F	3	1	0
CCP 2	58	F	3	0	0
IPMT 1	54	M	0	0	0
IPMT 2	77	M	3	0	0
NET	85	M	3	1	0
NET	76	F	3	1	0

^1^: in years; ^2^:F, female; M, male; ^3^:TNM stage: T, tumor; N, node; M, metastasis. PDACs, pancreatic adenocarcinomas; CCP, chronic calcific pancreatitis; IPMT, intraductal papillary or mucinous tumor; NET, neuroendocrine pancreatic tumor.

## DISCUSSION

MiRNAs have been shown to play essential role in regulating tumor progression, metastasis and response to radio- and chemo-therapy [14]. MiR-200 family is described to play anti-oncogenic functions and is frequently reported to be down-regulated in a variety of tumoral cells, including tumoral pancreatic cells [29–32]. However, as described for a number of cancers such as breast and ovarian cancers, conflicting data have been published reporting either under- or over-expression of different miR-200 family members in pancreatic cancers [29, 43–46]. Equally, evidence for both suppressive and oncogenic functions for miR-200s were provided [19, 30, 31, 45, 46]. A possible explanation for these conflicting reports is that elevated miR-200 expression would be associated with primary tumors, while loss of miR-200 expression would be found in metastatic cells which have undergone EMT [47, 48].

In this study we show that pancreatic tumoral cells displayed different levels of miR-200 expression, depending on the cell lines examined. Moreover, growth response to GEM was shown to be associated with miR-200 expression level, suggesting that miR-200s could be involved in chemo-sensitivity. This was comforted by the fact that forced expression of miR-200s in deficient Mia-Paca2 and PANC1 cells improved cell growth response to GEM (Supplementary Figure 6). Increasing number of reports has described an association between chemo-responsiveness and EMT in a large variety of cancers, including PDACs. Thus, gene expression profiling of chemoresistant tumoral pancreatic cell lines has shown a strong association between chemotherapy resistance and expression of genes associated with EMT such as ZEB1, Slug, Snail and vimentin [40, 52, 53]. Moreover, inverse correlation between chemoresistance and the expression level of miR-200 family members has been described in a number of cancers such as PDACs [32, 33]. In agreement with these data, only the epithelial markers cadherin1/E-cadherin and EpCam were expressed in BxPC3, Soj-6 and HPDE cells (Supplementary Figure 7). Conversely, Mia-Paca2 and PANC1 cells were shown to express high levels of the mesenchymal markers cadherin2/N-cadherin, vimentin, ZEB1 and Snai1, but not cadherin1/E-cadherin and EpCam, highlighting the link between chemoresistance, EMT and miR-200 gene repression. The miR-200 family has been reported to reverse EMT, among other by direct targeting of cadherin-1/E-cadherin transcriptional repressors ZEB1 and ZEB2 [19, 54]. In agreement with these data, we show that over-expression of miR-200s in Mia-Paca2 and PANC1 cells induced decreased expression levels of the mesenchymal markers Snai1 and ZEB1, with a concomitant augmentation of the expression of the epithelial markers cadherin-1/E-cadherin and EpCam. These results confirm that miR-200s contribute to improving chemo-responsiveness of pancreatic tumoral cells through repression of EMT inducers. All together, our data agree with reports supporting the idea that miR-200s exert essential anti-oncogenic functions in PDACs [55, 56] and led us to examine the molecular mechanisms involved in the loss of miR-200 expression in a subset of tumoral pancreatic cells.

In a number of cases, miRNA dysregulation has been linked to changes in epigenetic regulation, such as the methylation status or histone modification of miRNA genes, resulting in alterations in their expression levels. Methylation of miR-200 promoters, in particular miR-200c,141 cluster, has been described in a variety of tumoral cells and shown to result in the loss of miR-200 precursor and mature forms in these cells [41]. Although histone modifications of miR-200b,a,429 promoter is more currently reported, promoter hyper-methylation has also been found in a variety of cancer cells [41, 57]. Unexpectedly, whereas only methylated miR-200 gene promoters were present in Mia-Paca2 and PANC1 cells, both primary and pre-mature miR-200s could be detected in these cells. Recent approaches that enable genome-wide studies of the methylome have shown that the position of the methylated CpG islands in the transcriptional unit influences their capacity to repress gene transcription [50]. Although we cannot exclude the possibility that promoter methylation pattern differs in miR-200 -deficient *vs* miR-200 -proficient cells, the presence of miR-200 precursors in Mia-Paca2 and PANC1 cells could also be due to the presence of low levels of unmethylated promoter, undetectable under our experimental conditions. Intriguingly, whereas 5-AZA increased the expression of primary and pre-mature miR-200s, it failed to augment the level of mature miR-200s in Mia-Paca2 and PANC1 cells. Taken together, these data indicate that promoter hypermethylation is not sufficient to explain the deficit of mature miR-200s in these cells and strongly suggest that additional or alternative mechanisms are involved in miR-200 deficiency observed in Mia-Paca2 and PANC1 cells.

In the nuclear compartment, the ribonuclease Drosha cleaves pri-miRNAs to generate 70–100 nucleotide long pre-mature miRNAs (pre-miRNAs) which are then exported into cytoplasm where they undergo maturation process [4]. In this study, we show that pre-miR200s were detectable in nuclear but not cytoplasmic fractions of Mia-Paca2 and PANC1 cells, suggesting either a default in molecular mechanism involved in pre-miRNA nuclear export, or their rapid degradation by cytoplasmic ribonucleases. Pre-miRNAs are exported into cytoplasmic compartment by nuclear transporters, in particular Exportin 5 (XPO_5_) [52]. XPO_5_ also serves to protect pre-miRNAs from digestion by nucleases in the nucleus and during their export. Thus, deficiency in XPO_5_ expression or activity, may lead to the retention and degradation of pre-miRNAs in nuclear compartment [53]. Moreover, although minimal region in XPO_5_ is required for pre-miRNA binding, mutations in the XPO_5_ gene (exon 32) have been found in cell lines and primary tumors and shown to prevent XPO_5_ association with pre-miRNAs [32, 53]. However, we were able to co-immunoprecipitate XPO_5_ with pre-miR200s in nuclear fractions of both BxPC3 and Mia-Paca2 cells confirming that XPO_5_ is able to bind pre-miR-200s in mature miR-200 -proficient as well as -deficient cells. Transport of pre-miRNA/XPO_5_ complexes from nuclei to cytoplasm requires the action of the cofactor RanGTP that is hydrolyzed in RanGDP in cytoplasm, leading to the dissociation of the complex [33]. When Mia-Paca2 cells were treated with the non-hydrolysable RanQ69L-GTP, pre-miR-200s could be recovered by immunoprecipitation of XPO_5_ in cytoplasm of these cells, indicating that XPO_5_ is not only able to associate with pre-miR-200s in Mia-Paca2 cell nuclei, it is also able to export pre-miR-200s towards the cytoplasmic compartment. All together, these data demonstrate that the absence of miR-200s in cytoplasm of Mia-Paca2 cells is not due to impaired function of XPO_5_.

When exported into the cytoplasmic compartment, pre-miRNAs are further cleaved by the RNAse III enzyme Dicer, generating mature single strand miRNAs [12]. Reduced Dicer1 expression levels have been found in human tumors [45, 46]. Moreover*,* low *Dicer1* expression level was reported and shown to be associated with poor prognostic in a variety of tumors such as lung, breast and ovarian cancers, among others [45, 46, 58]. Interestingly, Illiou *et al.* [58] have described a down-regulation of miR-200 members in Dicer1 shRNA-depleted colonic cancer cells HCT16. Moreover, they observed that primary colorectal tumors with low expression of Dicer1 displayed reduced expression of miR-200s. In agreement with these data we show that mature miR-200 deficient Mia-Paca2 and PANC1 cells expressed low Dicer1 mRNA and protein levels, when compared to miR-200 proficient BxPC3 and Soj-6 cells. However, if the decrease in Dicer1 expression may indeed account for the loss or reduction of mature miR-200 forms, it seems unlikely that it is directly responsible for the absence of detectable pre-mature forms in cytoplasm of Mia-Paca2 and PANC1 cells. MiRNA biogenesis also depends on complex post-transcriptional processing that is controlled by many factors, including MCPiP1 [47]. MCPiP1 is a ribonuclease that acts as a broad suppressor of miRNA activity and biogenesis. MCPiP1 competes with Dicer1 in miRNA processing and has been described to suppress miRNA biosynthesis *via* cleavage of the terminal loops of pre-miRNAs [48]. The balance between Dicer-mediated processing and MCPiP1-mediated degradation affects miRNA biogenesis and potentially influences the normal and pathological miRNA regulation [59]. In this context, the possible involvement of MCPiP1 in the loss of mature miR-200s in Mia-Paca2 and PANC1 cells was then examined. Whereas MCPiP1 protein level was similar in all cell lines examined, Dicer1 level appeared much lower in miR-200 deficient Mia-Paca2 and PANC1 cells, when compared to miR-200 proficient BxPC3 and Soj-6 cells. These data suggested that MCPiP1 level might be sufficient to efficiently compete with Dicer1 for pre-miR-200 binding in Mia-Paca2 and PANC1 cells. However, co-immunoprecipitation analysis did not enable to demonstrate an association of pre-miR-200s with MCPiP1 in cytoplasmic fractions of Mia-Paca2 and PANC1 cells, possibly due to the rapid degradation of pre-miR-200s that prevents their detection. Nevertheless, inhibition of MCPiP1 expression in Mia-Paca2 and PANC1 cells resulted in a moderate but significant increase of detectable pre-mature as well as mature miR-200s in cytoplasmic compartment, confirming the involvement of MCPiP1 in the loss of miR-200s in these cells. Additionally, whether MCPiP1/Dicer1 ratio determines the fate of pre-miR-200s, increasing Dicer1 protein level was thought to allow the restoration of mature miR-200 expression in Mia-Paca2 and PANC1 cells. Over-expression of Dicer1 in Mia-Paca2 resulted in the reversion of MCPiP1/Dicer1 ratio, in favor of Dicer1. As expected, detectable pre-miR-200 amounts could be co-immunoprecipited with Dicer1 in cytoplasm of Dicer1-overexpressing Mia-Paca2 cells, and this was associated with a significant increase of the level of detectable mature miR-200s. These data demonstrate that the protein ratio MCPiP1/Dicer1 may be determinant in regulating miR-200 maturation process in a variety of tumoral pancreatic cells. Contrary to miR-200s, the levels of both precursor and mature forms of the miRNA miR-21 did not seem to be impacted by the high MCPiP1/Dicer1 ratio observed in Mia-Paca2 and PANC1 cells (data not shown). Heterogeneous effects of MCPiP1 knock-down on individual mature miRNAs have been previously reported and might be attributable to intrinsic properties of MCPIP1, as the action of MCPiP1 can be influenced by preferential target structures/sequences [48]. Additionally, specific partner protein(s) might modulate the activity of MCPiP1. Thus, several RNA-binding proteins such as Lin28, hnRNPA1 and KSRP interact with the terminal loop of miRNA precursors and are able to regulate the processing of pri-miRNAs and/or pre-miRNAs [60]. Considering that MCPiP1 also targets the terminal loop of pre-miRNAs, expression level of these RNA-binding proteins might influence MCPiP1 activity, in particular under conditions of low miRNA transcription level, as observed for miR-200s in Mia-Paca2 and PANC1 cells.

In order to evaluate the clinical relevance of our data, the potential association between miR-200 expression and MCPiP1/Dicer1 ratio was examined in a panel of malignant (PDACs) and non-malignant (non-MPDs) pancreatic tissues. In spite of the restricted number of samples, and in agreement with clinical studies describing down-regulation of miR-200 family members in tumoral pancreatic tissue [20, 30], PDACs were shown to express lower miR-141 and miR-429 levels than non-MPDs. Since miRNA maturation process is dependent, at least in part, on Dicer1 expression level and activity, we expected lower Dicer1 expression in PDACs than in non-MPDs. However, the mean level of Dicer1 was not significantly different in PDACs *vs* non-MPDs, suggesting that variations in its expression was not sufficient, *per se*, to explain the low level of miR-200s observed in PDACs. Although the difference in MCPiP1 expression between PDACs and non-MPDs appeared not significant, data reported in Figure 9 show that PDACs tissues tended to express higher MCPiP1 levels, when compared to non-MPDs (*P* = 0.065). Furthermore, the ratio MCPiP1/Dicer1 was found to be significantly higher in PDACs group than in non-MPDs group, strongly suggesting that MCPiP1 may interfere with miR-200 maturation process when present in excess over Dicer1. Although the analysis of a large cohort of tissue samples is required to definitively establish a correlation between MCPiP1/Dicer1 ratio and the level of miR-200s in pancreatic tissue, these data comfort the hypothesis that the loss of mature miR-200s in a subset of PDACs may depend on the relative expression of MCPiP1 and Dicer1.

MiRNAs are known to play a critical role in the initiation, progression and regulation of EMT in a variety of tumors including pancreatic cancers. Our present data agree with a number of studies describing anti-oncogenic functions for miR-200 family members. Thus, we confirm their role in the regulation of EMT process and chemotherapeutic response in a subset of tumoral pancreatic cells. Furthermore, we show that at least two mechanisms co-exist (epigenetic modification and activity of MCPiP1/Dicer1) and contribute to the repression of miR-200s in tumoral pancreatic cells. The observation that the level of mature, functional miR-200s may be impacted by the MCPiP1/Dicer1 ratio provides new insight into the mechanism responsible of the loss of miR-200s, and consequently of tumor progression and chemoresistance in pancreatic cancers.

## MATERIALS AND METHODS

### Materials

Cell culture medium and reagents, oligofectamine, lipofectamine 2000, random hexamers, turbo DNA-free kit, miRNA assay kit, miRNA reverse transcription kit, synthetic mature and pre-mature miRNAs, anti-miR-200 oligonucleotides, MCPiP1 siRNA, PCR TaqMan probes, Go TaqH hot start DNA polymerase and Moloney Murine Leukemia Virus Reverse Transcriptase (MMLV) were provided by Life Technologies. Gemcitabine (GEM), 5-Azacytidine (5-AZA) and anti-β-actine antibody were from Sigma-Aldrich. Enhanced chemiluminescent substrate was from Millipore. Protease inhibitor cocktail was provided by Roche Life Science. Kapa Fast Sybr qPCR Mix was from Clinisciences. RNAzol-RT, RanQ69L-GTP and anti-Dicer1 antibody were from Euromedex. GoTaq G2 DNA polymerase, Go Script reverse transcriptase and RNAsin RNase inhibitor were from Promega. PCR, qPCR and RT specific primers were obtained from Eurogentec. PolyA polymerase tailing kit, anti-MCPiP1, anti-Histone H1 and anti-calnexin antibodies were from Tebu-Bio. Anti-XPO_5_ antibody was from Cell Signalling. Protein A/G plus agarose was provided by Santa Cruz. pDestmycDICER1 expression vector was a gift from Thomas Tuschi (Addgene plasmid # 19873).

### Cell line culture

Human tumoral pancreatic cell lines BxPC3, Mia-Paca2, PANC1 and Capan-2 were obtained from the American Type Culture Collection (ATCC) (Rockville, MD). Soj-6 cell line was a gift from Dr M-J Escribano (INSERM UMR 911, Marseille, France) [49]. Normal pancreatic epithelial cells immortalized with SV40 (HPDE) was from ATCC. Mia-Paca2 and PANC1 cells were cultured in DMEM medium supplemented with 2 mM glutamine and 10% fetal bovine serum (FBS). Capan-2, BxPC3 and Soj-6 cell lines were maintained in RPMI medium with 10% FBS. HPDE cells were grown in Keratinocyte serum free-medium, supplemented with 5 ng/ml epidermal growth factor and 50 μg/ml bovine pituitary extract. Cells were cultured at 37° C, under a humid atmosphere of 5% CO_2_/95% air. Cells were regularly controlled for mycoplasma contamination and used for 10–15 passages from the passage of origin. KRAS mutations were examined for each cell line as described in Supplementary Materials and Methods and data reported in Supplementary Table 1.

### Human pancreatic tissues

Tissues samples were from the CRO2 tissues collection (agreement DC-2013-1857). All the tissue protocols were approved by the relevant institutional committees (Aix-Marseille University) and were undertaken under informed consent of each patient or relatives. All the samples were anonymized, and postsurgical anatomopathology reports were provided. Tumor tissues samples were obtained after pancreatic resection (duodeno-pancreatectomy) from 6 patients diagnosed with pancreatic adenocarcinoma (PDACs) (Gastroenterology and Digestive Surgery departments of different Marseille hospitals, France) between 2008 and 2016. A definitive diagnosis of pancreatic adenocarcinoma was established after histochemical analysis of the resected tumor tissues. WHO and TNM stages were determined by a senior pathologist. Tissue samples were collected from patients who had received no previous local or systemic treatment prior to surgery. Patients with non-malignant pancreatic diseases (*n* = 6) constituted the non-malignant pancreatic diseases (non-MPDs) group. Patient clinical data are reported in Table 4.

### Preparation of cell extracts and western immunobloting

Total cell lysates, cytoplasmic and nuclear extracts were prepared as previously described [50]. The quality of nuclear and cytoplasmic preparations was verified by immnoblotting of Histone H1 and calnexin, respectively. Cell extracts were resolved by SDS-PAGE and separated proteins were then electroblotted on PVDF membrane. Immunoblotting was performed as previously reported [50]. Quantification of the immunoblots was performed by image analysis using the Macintosh-based public domain program Image (ImageJ software, https://imagej.nih.gov/ij).

### Immunoprecipitation

Cell extracts prepared as described above were 5 fold diluted in ice-cold PBS. Antibodies (1–2 μg) were added to 1 ml of cell extract. After incubation for 16 h at 4° C, immuno-complexes were precipitated for 2 h at 4° C with protein A/G plus agarose as indicated by the supplier. Immunoprecipitates were washed 5 folds with ice-cold PBS, separated by electrophoresis on SDS-acrylamide gels then electroblotted on PVDF membrane. In some cases, immunoprecipitates were submitted to RNA extraction.

### RNA extraction and reverse transcriptase polymerase chain reaction (RT-PCR)

RNA was prepared with RNAzol-RT as specified by the manufacturer. For the preparation of RNA from pancreatic tissues, samples were first fragmented into small pieces then total RNA was extracted with 1 ml of RNAzol-RT per 100 mg of frozen tissue. RNA samples were treated with DNAse I, using Turbo DNA-free kit. RNA purity was assessed by spectrophotometric measurement of the OD_260_/OD_280_ and OD_260_/OD_230_ ratios with acceptable values falling between 1.8 and 2.1. RNA integrity was verified by electrophoresis on 1% agarose gel. RT-PCR was performed as previously described [22] and PCR products were separated on agarose gels. SYBR Green real-time PCR (RT-qPCR) for the genes of interest was performed using Kapa Sybr Fast PCR mix. Amplification and analysis was done on a LightCycler 480 apparatus (Roche Applied Science). The relative expression levels were calculated using the comparative Ct method (∆∆Ct). Eukaryotic 28s rRNA housekeeping gene was used for data normalization. Sequences of the primers used are reported in Supplementary Table 2.

### Measurement of pre-mature and mature miRNAs by quantitative real-time polymerase chain reaction (RT-qPCR)

Specific quantitative real-time PCR experiments for mature microRNAs were carried out on a total of 40 ng RNA, using TaqMan MicroRNA assay as indicated by the manufacturer. Amplification was followed by real-time PCR on a Roche LightCycler 480. Gene expression was normalized according to the expression of the small nuclear U6 gene (snU6).

The expression of pre-mature miR-200s (pre-miR-200s) was assessed by the S-poly(T) method, as described by Kang *et al* [51]. Briefly, 1 μg total RNA was polyadenylated with Poly(A) Polymerase Tailing kit, following the instructions of the manufacturer. Reverse transcription was performed in a 10 μl reaction mix containing 1 μl of the polyadenylation reaction product, 1 μl of 0.5 μM S-poly(T) RT primer, 0.5 μl of 10 mM dNTP, 1 μl of MMLV buffer 10×, and 50 units of MMLV. The reaction was incubated at 42° C for 60 min, then stopped by heating at 85° C for 5 min. The RT products were amplified and detected using a universal Taqman probe. PCR reaction (20 μl total) contained 0.5 μl of RT products, 4 μl of colorless GoTaqH flexi buffer 5×, 0.5 unit of GoTaqH Hot Start Polymerase, 1.5 mM MgCl_2_, 0.2 mM dNTP, 0.2 μM forward primer, 0.2 μM universal reverse primer, and 0.25 μM universal Taqman probe. Oligonucleotide sequences used in this study are reported in Supplementary Table 2.

### Cell transfection

Three successive cycles of transfection were performed for transfecting cells with synthetic miR-200 oligonucleotides at 35 nM, final concentration. Transfection was performed in serum-free medium, using oligofectamine transfection reagent and following the instructions of the manufacturers. In parallel, control cells were transfected with control miRNA oligonucleotides. After 24 hours, the medium was replaced with fresh medium containing 10% serum. Gene expression was evaluated 48 hours post-transfection. To evaluate the effects of the transfection on cell proliferation, cells were allowed to grow for additional 96 hours in the absence or in the presence of GEM as indicated. To examine the role of MCPiP1 in the loss of mature miR-200s in Mia-Paca2 and PANC1 cells, specific siRNA was used to block MCPiP1 expression. Cells were transfected as described above, with 50 nM MCPiP1 siRNA and in the presence of oligofectamine. Control cells were transfected with no relevant siRNA oligonucleotide. Gene expression was measured 48 hours following the transfection.

### MCPiP1 ribonuclease activity assay

In order to examine the ability of MCPiP1 to degrade pre-miR-200s, MCPiP1 was immunoprecipitated from Mia-Paca2 cell lysate as described above. Immunoprecipitated MCPiP1 was then incubated in the presence of pre-miR-200 oligonucleotides (10 pg) in RNA cleavage buffer (25 mM Hepes, 50 mM potassium acetate, 5 mM DTT, 5 mM MgCl_2_, 0.5 unit RNAsin) for 30 min at 30° C. In parallel, immunoprecipitation was done with no relevant IgG as control. At the issue of the incubation, RNA was extracted as described above and remaining pre-miRNAs were quantified by RT-qPCR by means of a standard curve established in parallel with 0.5–20 pg synthetic pre-miR-200 oligonucleotides.

### Statistical analysis

The Graph Prism version 6.0 software (https://www.graphpad.com/scientific-software/prism/) was used to generate statistical data. Statistical analyses were evaluated using the non-parametric Mann–Whitney *U*-test between two groups. Significance was accepted at *P* ≤ 0.05.

## SUPPLEMENTARY MATERIALS FIGURES AND TABLES


