# Supplementary material for: Ribonuclease MCPiP1 contributes to the loss of micro-RNA-200 family members in pancreatic cancer cells
Source: Oncotarget. 2018 Nov 13;9(89):35941–61. doi: 10.18632/oncotarget.26310 (PMC6267598; doi:10.18632/oncotarget.26310)
Supplement: Supplementary file 1 [file oncotarget-09-35941-s001.pdf]

Ribonuclease MCPiP1 contributes to the loss of micro-RNA-200 family members in pancreatic cancer cells

SUPPLEMENTARY MATERIALS

MATERIALS AND METHODS

Cell growth assay

Cells were plated in 24 well-plates (10,000–20,000 cells/well). Two days later, culture medium was renewed and cells received different concentrations of GEM for 5 days. Control cells were treated in the presence of the diluent. Cell culture medium and treatment were renewed every two days. At the end of the treatment, cell proliferation was assessed with MTT assay (Sigma-Aldrich). Optical density was measured at 570 nm, with background subtraction at 690 nm.

Methylation-specific PCR analysis for miR-200 gene promoters

Genomic DNA was prepared with Wizard Genomic DNA purification kit 5 (Promega) as described by the manufacturer, then 1 mg DNA was converted by using the EpiTect bisulfite kit (Qiagen) and eluted in 20 µl buffer. Methylation-specific PCR (MSP) analyses for methylation of 5′-CpG- rich sequences in the promoters of miR-200b,a,429 and miR-200c,141 genes were performed by using primers specific for the unmethylated or methylated promoter sequences. Briefly, 1 µl sodium bisulfite-modified DNA was amplified in a total of 10 µl containing 1 ml buffer 10×, 200 mM dNTPs, 0.6 µM of each primers and 0.25 U GoTaqH Hot start polymerase (Life Technologies). PCR products were analyzed on 2% agarose gel. Specific primers were designed with Methylprimers Express software and their sequence reported in Supplementary Table 3.

Analysis of KRAS mutations

KRAS mutations were analyzed for each cell lines as previously described (Tomasini *et al*, Int. J. Mol. Sci. 2016). Briefly, Genomic DNA was extracted using NucleoSpin DNA kit (Macherey-Nagel, Düren, Germany) according to the manufacturer's protocol. PCR amplification and HRM analysis from genomic DNA were carried out on LightCycler® 480 (Roche Diagnostics, Meylan, France). Wild-type DNA (placenta), and PCR negative controls were included as control. Temperature ramping and fluorescence acquisition settings were recommended by the manufacturer. HRM curves were normalized for each sample, and compared with wild-type genomic DNA using LightCycler® 480 gene-scanning software. Sanger sequencing was performed after DNA purification using Big Dye Terminator v3.1 cycle sequencing kit (Life Technologies, Villebon-sur-Yvette, France) on Evo75® (Tecan, Männedorf, Switzerland). Sequences were analyzed on 3500 or 3130 Dx Genetic Analyser® (Applied Biosystems, Villebon-sur-Yvette, France).

Supplementary Table 1: Analysis of KRAS mutations in pancreatic cell lines

Cell lines	KRAS
Mia-Paca2	G12C
PANC1	G12N
BxPC3	wt
Soj-6	G12V
Capan-2	G12V
HPDE	wt

Wt, wild type.

Supplementary Table 2: Sequence of primers used for RT-PCR and RT-qPCR analysis

	qPCR primers
pri-miR-200b,a,429 **FW**	5'-GCT GCT CGT TGG CTT TAC AG-3'
pri-miR-200b,a,429 **RV**	5'-AGG CTC ATC AGT CAT TGC GT-3'
pri-miR-200c,141 **FW**	5'-GCC TTA AAG CCC CTT CGT CT-3'
pri-miR-200c,141 **RV**	5'-ACA CAC CGA TTT ACC CAC CC-3'
pre-miR-429 S-Poly(T) **RT primer**	5'-CAG TGC AGG GTC CGA GGT CAG AGC CAG CTG GGC AAT TTT TTT TTT TTG CAG CG-3'
pre-miR-141 S-Poly(T) **RT primer**	5'-CAG TGC AGG GTC CGA GGT CAG AGC CAG CTG GGC AAT TTT TTT TTT TTG AAC CC-3'
pre-miR-429 **FW**	5'-CTC TGT CTA ATA CTG TCT GG-3'
pre-miR-141 **FW**	5'-CCA GTA CAG TGT TGG ATG GTC TAA-3'
Universal **RV**	5'-CAG TGC AGG GTC CGA GGT-3'
Taqman probe	5'-6-FAM-CAG AGC CAC CTG GGC AAT TT-MGB-3'
Cadherin1/E-cadherin **FW**	5'-GCC GAG AGC TAC ACG TTC A-3'
Cadherin1/E-cadherin **RV**	5'-GAC CGG TGC AAT CTT CAA A-3'
EpCam **FW**	5'-GCC GCA GCT CAG GAA GAA T-3'
EpCam **RV**	5'-TGA AGT ACA CTG GCA TTG ACG AT-3'
DICER1 **FW**	5'-TTC AGA AAA CAG GAA GAG GTAC T-3'
DICER1 **RV**	5'-TTA GAG ATG GGT GCC CTT GC-3'
MCPiP1 **FW**	5'-ACG ACA CAT ACC GTG ACC TC-3'
MCPiP1 **RV**	5'-GGG GGC ATA AAC TTG TCA TTG-3'
Cadherin2/N-cadherin **FW**	5'-GACAATGCCCCTCAAGTGTT-3'
Cadherin2/N-cadherin **RV**	5'-CCATTAAGCCGAGTGATGGT-3'
SNAI1 **FW**	5'-ACC ACT ATG CCG CGC TCT T-3'
SNAI1 **RV**	5'-TAG GGC TGC TGG AAG GTA AAC TC-3'
Vimentin **FW**	5'-AAA GTG TGG CTG CCA AGA AC-3'
Vimentin **RV**	5'-AGC CTC CAG AGA GGT CAG CAA-3'
ZEB1 **FW**	5'-GAA AGT GAT CCA GCC AAA TGG-3'
ZEB1 **RV**	5'-GGC GGT GTA GAA TCA GAG TCA TTC-3'
28S **FW**	5'-CAG CCA AGC TCA GCG CAA C-3'
28S **RV**	5'-AGC CGA TCC ATC ATC CGC AAT G-3'

FW, forward primers; RV, reverse primers; RT primer, reverse transcription specific primer.

Supplementary Table 3: List of primers used for DNA methylation analysis

Promoters	Primer sequences
miR-200b,a,429 (**M)**	FW : 5′-GGGTCGGGAAAGGTTGTC-3′
	RV : 5′-CACCCTAAATCGCTAATCACGA-3′
miR-200b,a,429 (**UM)**	FW : 5′-GGGTTGGAAAGGTTGTT-3
	RV : 5′-CACCCTAAATCACTAATCACAA-3′
miR-200c,141 (**M)**	FW : 5′-CGTTATCGTTATTGGTTC-3′
	RV : 5′-CACAAATACGAACTCCCG-3′
miR-200c,141 (**UM)**	FW : 5′-TTGTGTTATTGTTATTGGTTT-3′
	RV : 5′-CACAAATACAAACTCCCACTA-3′

M, methylated ; UM, unmethylated. FW, forward primers ; RV, reverse primers.

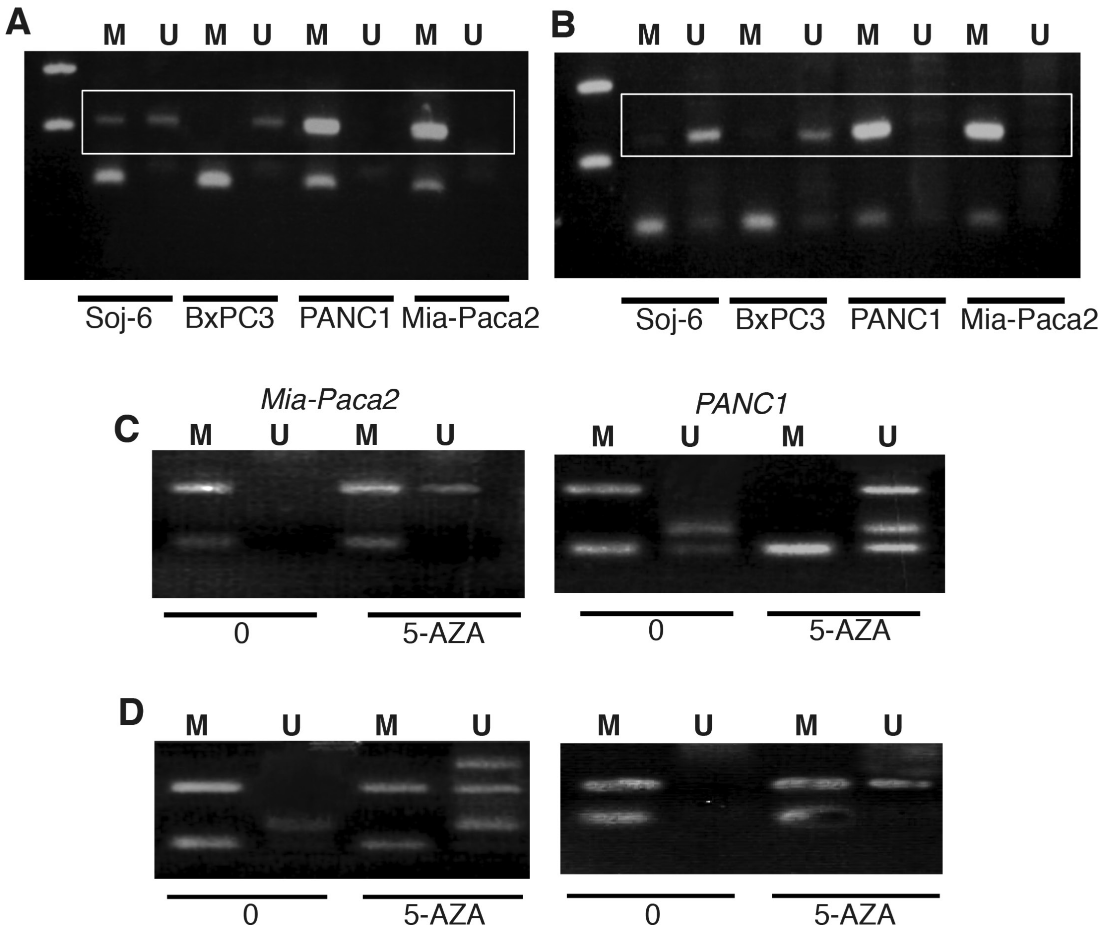

Supplementary Figure 1: Methylation analysis of miR-200 gene promoters. Methylation status of miR-200b,a,429 (**A**) and miR-200c,141 (**B**) gene promoters was examined by methylation-specific PCR on DNA extracted from tumoral pancreatic cell lines. The effect of the demethylant agent 5-AZA on miR-200b,a,429 (**C**) and miR-200c,141 (**D**) promoter methylation was analyzed by treating Mia-Paca2 and PANC1 cells for 2 days with 10 μM 5-AZA. Following methylation specific PCR, PCR products were separated on 2% agarose gel. Data are representative of 2 separate experiments. U, unmethylated; M, methylated.

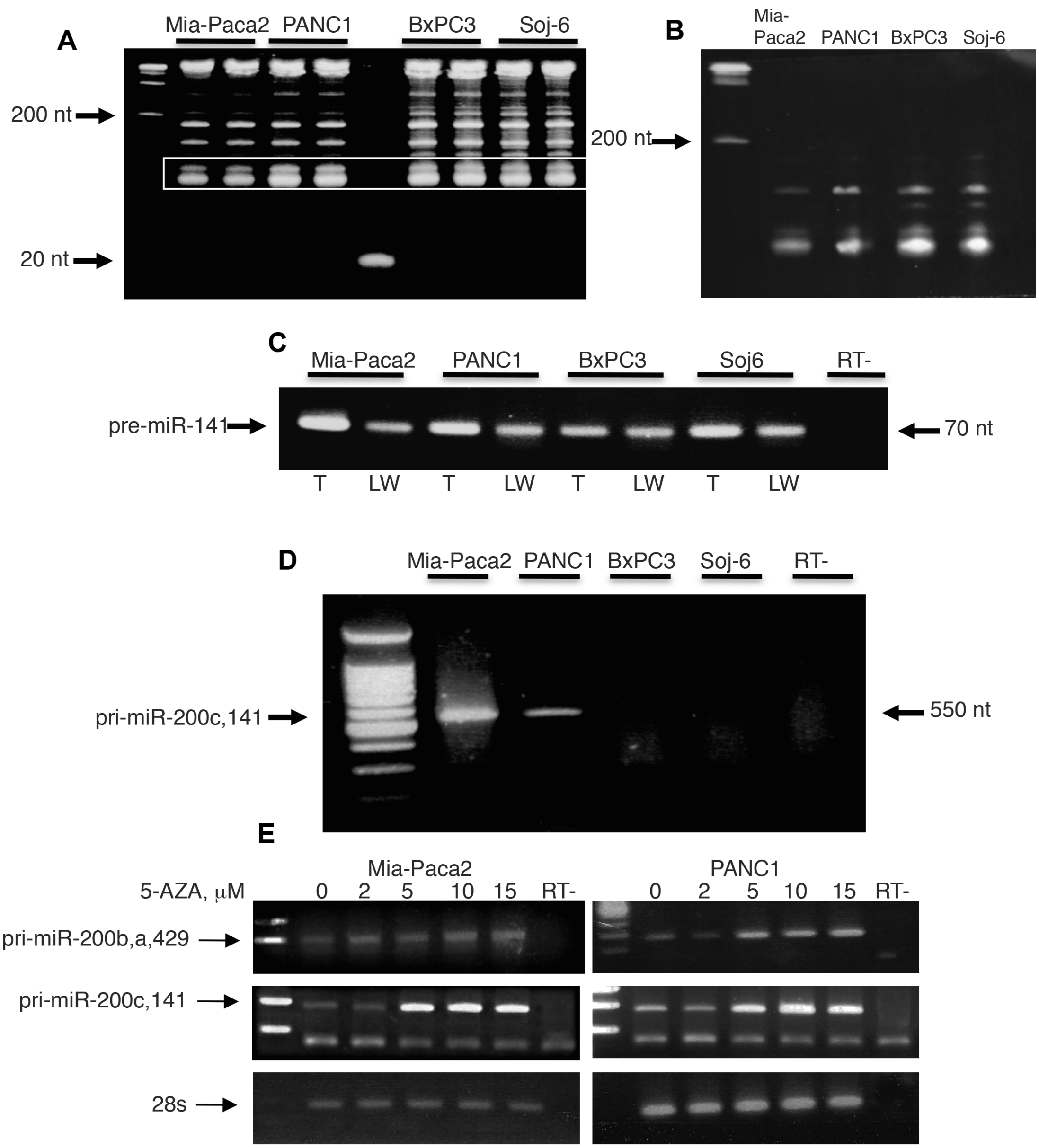

Supplementary Figure 2: PCR amplification of pre-mature miR-200s in low molecular weight RNA preparations. (**A**), total RNA extracted from tumoral pancreatic cell lines was separated on 8% acrylamide gel. (**B**), RNA bands below 200 nt (LW) were extracted then further separated on 12% acrylamide gel to verify the quality of the preparation. (**C**), the levels of pre-mature miR-141 were determined by RT-PCR on LW-RNAs. (**D**), primary miR-200c,141 was amplified by RT-PCR in both LW and total RNA. (**E**), Primary miR-200s were amplified by RT-PCR in Mia-Paca2 and PANC1 cells treated with the indicated concentrations of 5-AZA. T, total RNA; LW, low molecular weight RNA; pre-miR-141, pre-mature miR-141; pri-miR-200c,141; primary miR-200c,141.

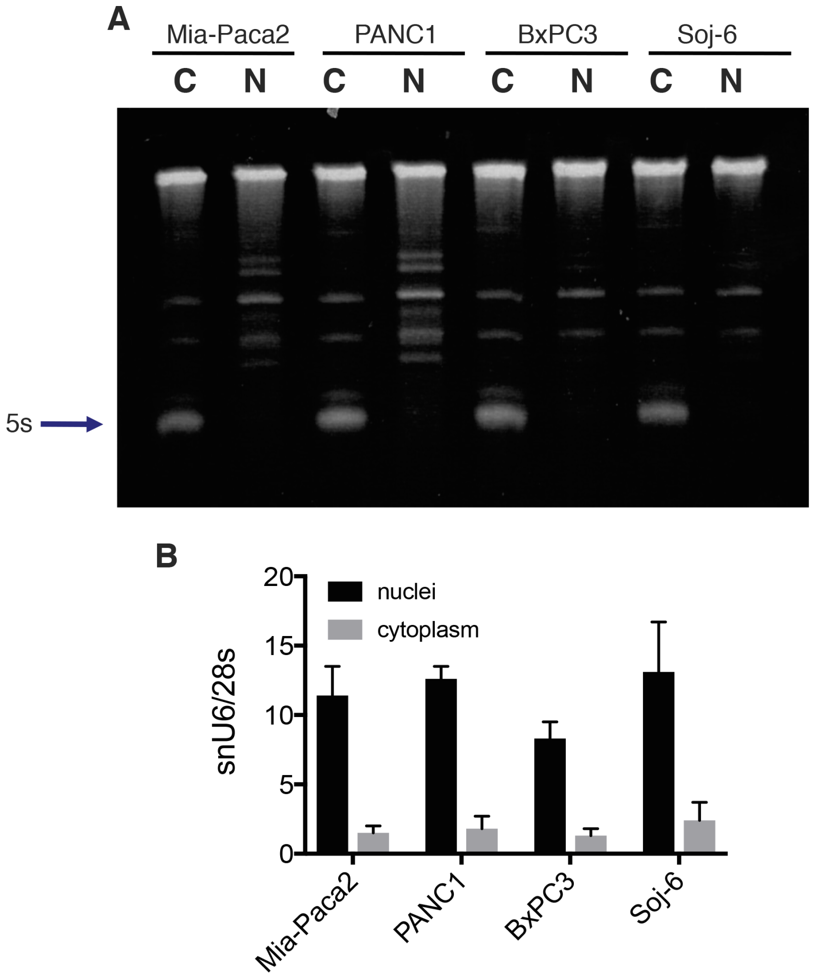

Supplementary Figure 3: Preparation of RNA from nuclear and cytoplasmic fractions of tumoral pancreatic cell lines. Nuclear (N) and cytoplasmic (C) fractions were prepared from tumoral pancreatic cell lines. (**A**), total RNA was extracted from both subcellular fractions then separated by electrophoresis on 8% acrylamide gel. The presence of the 5s ribosomal RNA is specific of the cytoplasmic compartment. (**B**), purity of the RNA preparations was further assessed by the measure of the expression of small nuclear U6 (snU6) gene by RT-qPCR.

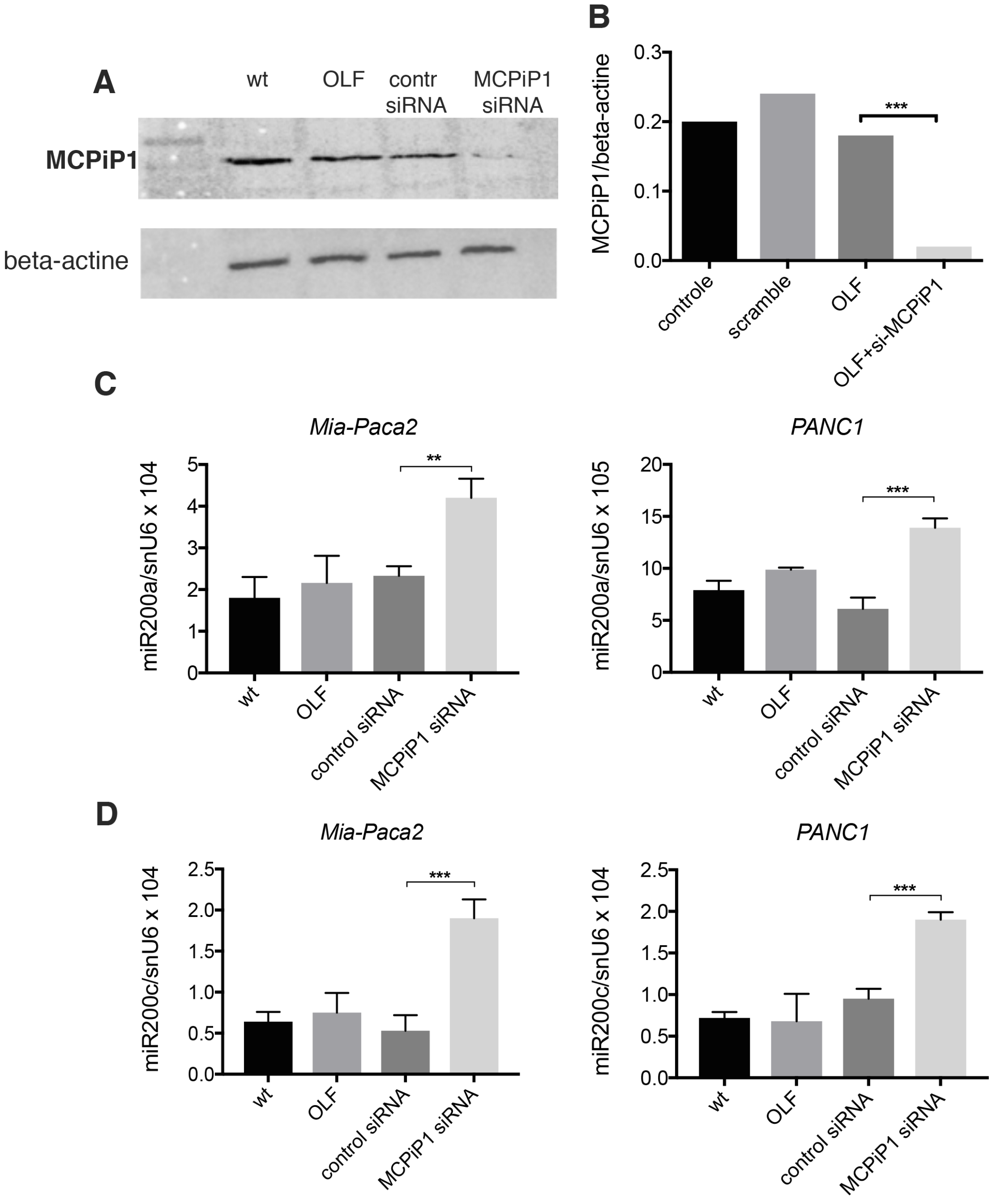

Supplementary Figure 4: Effect of MCPiP1 inhibition on miR-200 expression level in Mia-Paca2 and PANC1 cell lines. Mia-Paca2 and PANC1 cells were transfected with MCPiP1 siRNA in the presence of the transfection reagent oligofectamine. (**A**), two days post-transfection, MCPiP1 protein level was assessed by immunoblotting. (**B**), band intensity was quantified with ImageJ software and normalized with β-actin protein level. Expression levels of miR-200a (**C**) and miR-200c (**D**) were measured by RT-qPCR. wt, wild type cells; OLF, oligofectamine; contr siRNA, control siRNA. $^{**}P < 0.01$; $^{***}P < 0.001$.

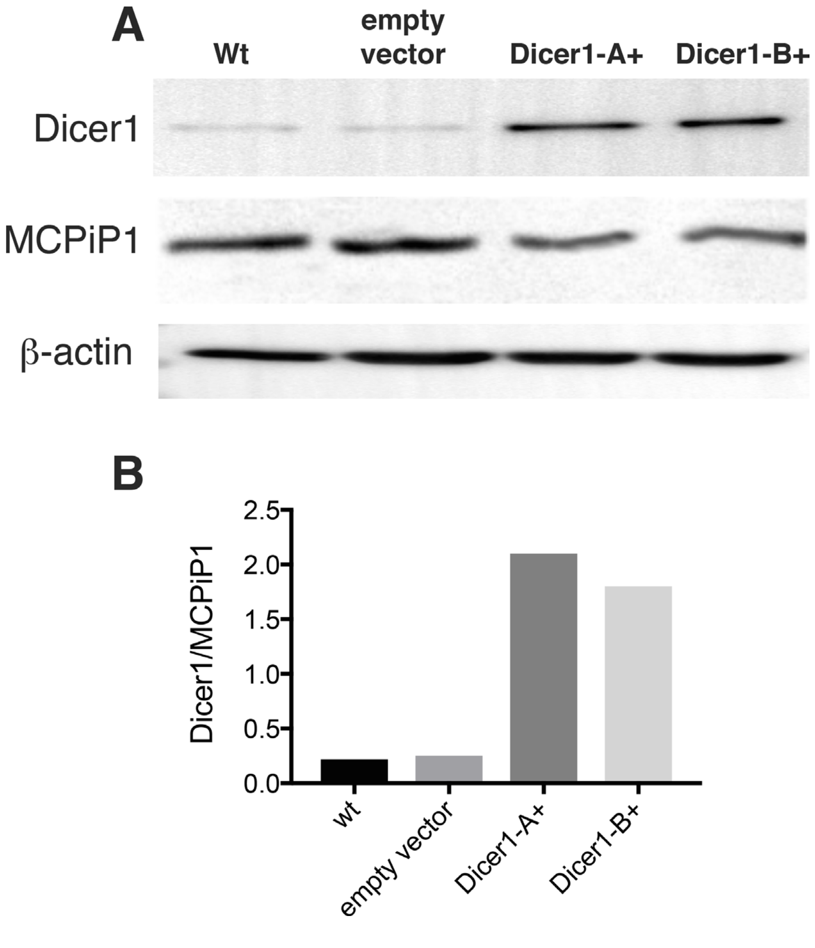

Supplementary Figure 5: Expression of Dicer1 and MCPiP1 in Dicer1-overexpressing Mia-Paca2 cell line. Mia-Paca2 cells were transfected with Dicer1 expressing vector, then two clones stably over-expressing Dicer1 were selected. Control cells were transfected with empty vector. (**A**), immunoblot analysis of Dicer1, MCPiP1 and β-actin in Mia-Paca2 cell lysates. (**B**), The integrated optical density of each band detected by immunoblotting was quantified using ImageJ software. Values obtained for each sample were normalized for the corresponding β-actin protein level, then protein expression ratio between MCPiP1 and Dicer1 was determined. Wt, wild type cells; Dicer1-A+ and Dicer1-B+, Dicer1 over-expressing clones.

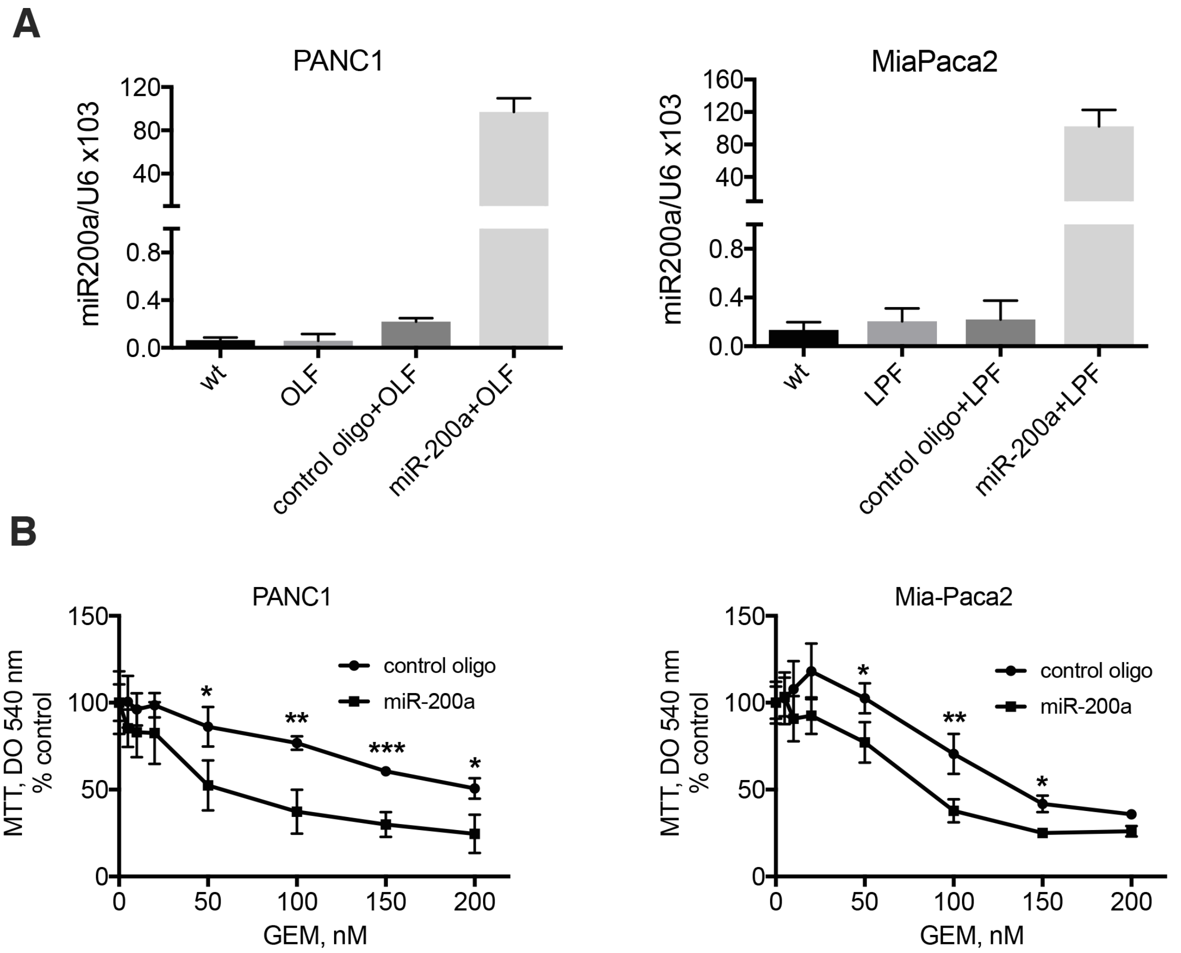

Supplementary Figure 6: Effect of miR-200 over-expression on gemcitabine (GEM) growth effect in PANC1 and Mia-Paca2 cell lines. (**A**), PANC1 and Mia-Paca2 cells were incubated for 24 h with transfection reagent in the absence or in the presence of 35 nM synthetic miR-200a oligonucleotide. Cells were then placed in medium containing 10% serum and allowed to grow for additional 24 h. Transfection was then renewed twice. RNA was extracted and miR-200a level was measured by RT-qPCR. Control cells were transfected with control miRNA oligonucleotide. (**B**), cells transfected with synthetic miR-200a oligonucleotide were treated for 5 days in the presence of the indicated concentrations of GEM. Culture medium and treatment were renewed every two days. Cell proliferation was then assessed by MTT metabolism. Control cells were transfected with control miRNA oligonucleotide. Values are expressed as % of GEM-untreated cells. Data are the mean ± SD of 3 separate experiments. Wt, wild type cells; OLF, oligofectamine transfection reagent; LPF, lipofectamine 2000 transfection reagent; control oligo, control miRNA oligonucleotide. $^{*}P < 0.05$; $^{**}P < 0.01$; $^{***}P < 0.001$.

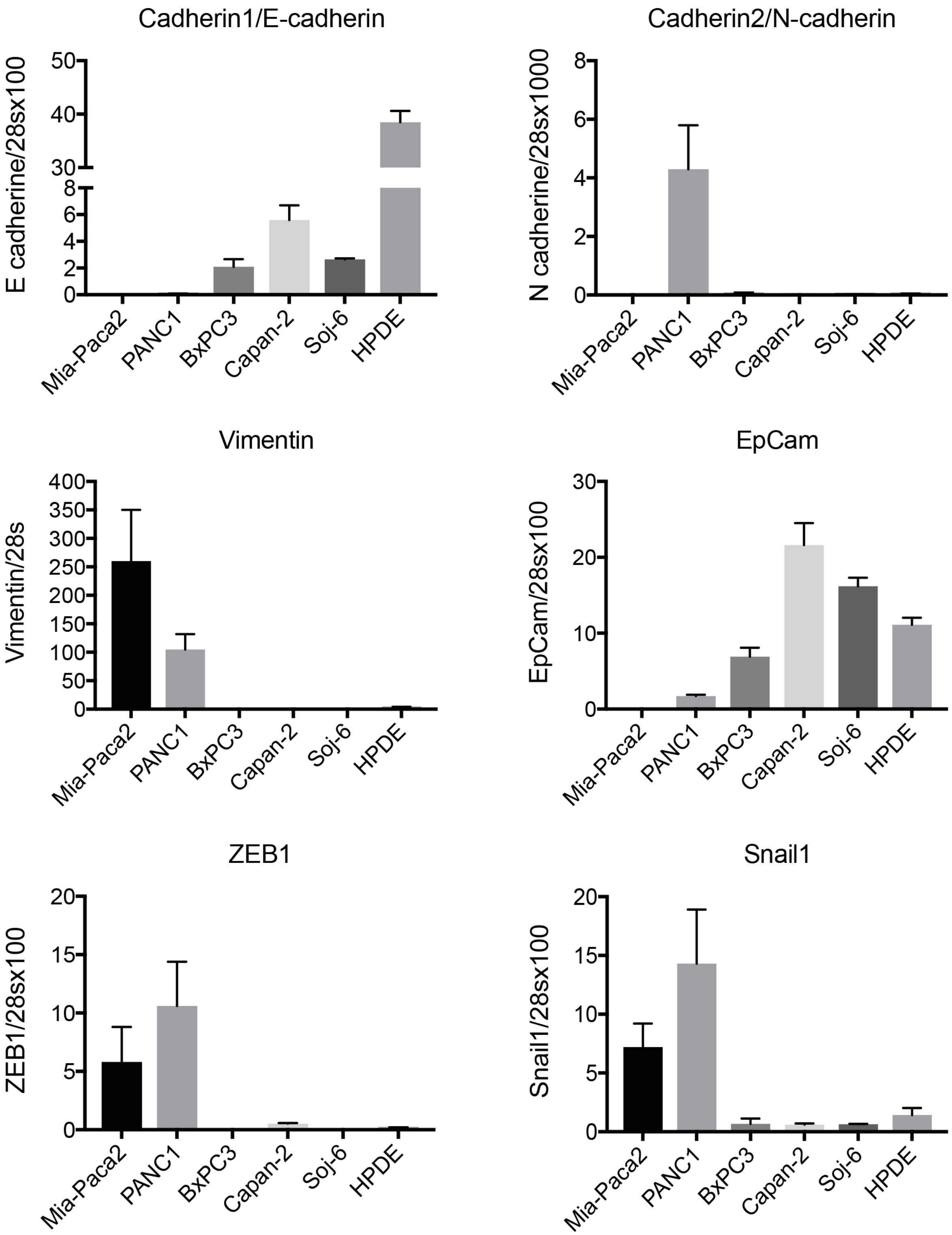

Supplementary Figure 7: Expression of mesemchymal and epithelial markers in tumoral pancreatic cell lines. Gene expression was determined by RT-qPCR on RNA extracted from tumoral pancreatic cells. Data were normalized with the expression of the housekeeping gene 28S ribosomic RNA.
